# Efficacy of psychological interventions for post-traumatic stress disorder in children and adolescents exposed to single versus multiple traumas: meta-analysis of randomised controlled trials

**DOI:** 10.1192/bjp.2023.24

**Published:** 2023-05

**Authors:** Thole H. Hoppen, Richard Meiser-Stedman, Tine K. Jensen, Marianne Skogbrott Birkeland, Nexhmedin Morina

**Affiliations:** Institute of Psychology, University of Münster, Münster, Germany; Department of Clinical Psychology and Psychological Therapies, Norwich Medical School, University of East Anglia, Norwich, UK; Department of Psychology, University of Oslo, Oslo, Norway; and Norwegian Centre of Violence and Traumatic Stress Studies, Oslo, Norway; Norwegian Centre of Violence and Traumatic Stress Studies, Oslo, Norway

**Keywords:** PTSD, psychological intervention, multiple trauma, efficacy, meta-analysis

## Abstract

**Background:**

Previous meta-analyses of psychotherapies for children and adolescents with post-traumatic stress disorder (PTSD) did not investigate whether treatment efficacy is diminished when patients report multiple (versus single) traumas.

**Aims:**

To examine whether efficacy of psychological interventions for paediatric PTSD is diminished when patients report multiple (versus single) traumas.

**Method:**

We systematically searched PsycInfo, MEDLINE, Web of Science and PTSDpubs on 21 April 2022 and included randomised controlled trials (RCTs) meeting the following criteria: (a) random allocation; (b) all participants presented with partial or full PTSD; (c) PTSD is the primary treatment focus; (d) sample mean age <19 years; (e) sample size *n* ≥ 20. Trauma frequency was analysed as a dichotomous (single versus ≥2 traumas) and continuous (mean number of exposures) potential moderator of efficacy.

**Results:**

Of the 57 eligible RCTs (*n* = 4295), 51 RCTs were included in quantitative analyses. Relative to passive control conditions, interventions were found effective for single-trauma-related PTSD (Hedges’ *g* = 1.09; 95% CI 0.70–1.48; *k* = 8 trials) and multiple-trauma-related PTSD (*g* = 1.11; 95% CI 0.74–1.47; *k* = 12). Psychotherapies were also more effective than active control conditions in reducing multiple-trauma-related PTSD. Comparison with active control conditions regarding single-event PTSD was not possible owing to scarcity (*k* = 1) of available trials. Efficacy did not differ with trauma exposure frequency irrespective of its operationalisation and subgroup analyses (e.g. trauma-focused cognitive–behavioural therapy only).

**Conclusions:**

The current evidence base suggests that psychological interventions for paediatric PTSD can effectively treat PTSD in populations reporting single and multiple traumas. Future trials for PTSD following single-event trauma need to involve active control conditions.

Post-traumatic stress disorder (PTSD) is a common disorder in children and adolescents.^1^ Meta-analytic reviews of randomised controlled trials (RCTs) have concluded that psychological interventions for PTSD produce large and medium effect sizes compared with waiting-list and active control conditions respectively.^2,3^ However, patients in these trials differed with respect to whether they had been exposed to single or multiple traumatic events. Research findings suggest that youth exposed to multiple traumatic events are more likely to develop PTSD and have more severe PTSD symptoms than those exposed to single traumatic events.^4–6^ Crucially, research further suggests that multiple exposure to traumatic events is associated with elevated lifetime adversity, greater likelihood of living in disordered communities and low levels of family support.^6^ This suggests that youth exposed to multiple traumas may gain less from current psychological interventions than youth with exposure to a single trauma. Existing meta-analytic reviews have not yet addressed this relevant patient characteristic as a potential moderator of treatment efficacy. Potential differences in treatment outcome for youth exposed to single versus multiple traumas would make adjustment of current treatments necessary to better meet their needs. Against this background, we aimed to examine whether treatment efficacy is diminished when patients report multiple (versus single) traumas. To this end, we conducted a systematic review and meta-analysis of RCTs on the efficacy of psychological interventions for paediatric PTSD.

## Method

The aims and methods of this meta-analysis were pre-registered with the PROSPERO database (registration number CRD42022338484) and Preferred Reporting Items for Systematic Reviews and Meta-Analysis (PRISMA) guidelines^7^ were followed. Two raters independently conducted the systematic literature search (title and abstract screening, full-text screening) and all following steps (data extractions, risk of bias assessment, categorisation of single versus multiple trauma trials). Disagreements were systematically analysed after each step and discussed among the authors until consensus was reached. We pre-registered the formulation of the main research question of the present work in terms of the Population, Intervention, Comparison, Outcome and Study (PICOS) framework as follows: in children and adolescents with full or partial PTSD (P), are psychological interventions (I), compared with passive control conditions, active control conditions or psychological interventions of another family of intervention (C), less effective when (most) participants survived multiple- versus single-event trauma (O), as studied in randomised controlled trials (S)?

### Identification and selection of studies

Trials were eligible if they fulfilled the following five inclusion criteria: (a) random group allocation; (b) all participants presented with partial or full PTSD at baseline; (c) PTSD was the primary treatment focus and outcome; (d) sample mean age <19.0 years; (e) sample size *n* ≥ 20. The last gives adequate power on a study level and thus excludes chance findings from underpowered studies.^8,9^ In line with the second and third inclusion criteria, we excluded trials with a (partial or full) preventive focus. In line with the third inclusion criterion, samples with comorbidities (i.e. the presence of other mental disorders) were only included when PTSD was the primary treatment focus and outcome. We excluded non-inferiority trials that compared only two arms from the same family of interventions.^10^

For the period from database inception up until 30 April 2019, we relied on our previous systematic search.^2^ For the period thereafter, we conducted a new systematic search on 21 April 2022 in the databases PsycInfo, MEDLINE, Web of Science and PTSDpubs using the same search terms. We conducted multi-field searches using search terms for PTSD (e.g. posttraumatic stress OR post-traumatic stress OR posttraumatic syndrome* OR PTSD OR PTSS) and treatment (e.g. treatment* OR intervention* OR therapy OR psychotherapy OR exposure OR trial OR counselling). No restrictions on language were applied. See Supplementary Appendix A, available at https://dx.doi.org/10.1192/bjp.2023.24 for the full search strategy. We further reviewed 41 related qualitative and quantitative reviews (see Supplementary Appendix B for their references) as well as the reference lists of all included trials.

### Risk of bias assessment

We rated the quality of trials by means of eight dichotomous quality criteria originally based on Cochrane Collaboration criteria to assess trial methodological validity^11^ and authoritative criteria for empirically supported psychological interventions^12^ originally reported in meta-analytic work conducted by Cuijpers and colleagues.^13^ These criteria have been widely used in meta-analytic research, including research on psychological treatment of PTSD.^2,14^ Trials received a positive quality score of 1 for each of the following criteria that held (giving a quality sum score ranging from 0 to 8): (a) all participants were diagnosed with PTSD on the basis of a (semi-)structured diagnostic interview; (b) the trial used and followed a treatment manual; (c) study therapists were formally trained to apply the given treatment manual as part of the study or study therapists had extensive experience in applying this particular treatment manual; (d) adherence to the treatment manual was formally checked via regular supervision or adherence ratings of recordings; (e) intention-to-treat (ITT) analyses were reported; (f) *n* ≥ 50 (i.e. *n_1_* + *n_2_* ≥ 50); (g) randomisation was truly random and random allocation was performed independently either by an independent party or a computer algorithm; and (h) outcome assessment was performed masked (‘blind’) either via masked assessors in diagnostic interviews or via self-report measures.

### Definition of single-event-trauma versus multiple-trauma trials

Trials exclusively involving participants who had reported a single event (i.e. exposure) of trauma were coded as single-event-trauma trials. Trials with samples in which 50–100% of the included participants had reported more than one traumatic event (i.e. two or more trauma exposures irrespective of trauma type) were categorised as multiple-trauma trials. Trials with samples in which 0–49% of the included participants had reported more than one traumatic event were not included in the analyses. The choice of the 50% cut-off for the multiple-trauma trials was chosen in an effort to balance out the number of trials per category and thus the statistical power of the main analyses as well as the internal validity of the main research question (i.e. making sure that most participants in the samples had survived multiple rather than single traumas). However, we also performed a sensitivity analysis with a more conservative definition of multiple-trauma trials (i.e. ≥90% of the sample with multiple lifetime exposures to traumatic events). Trials with insufficient reporting on trauma frequency could not be included in the quantitative synthesis.

### Categorisation of treatment and control groups

In line with our previous work,^2^ trial arms were first coded and categorised into either active treatment groups or control groups. The active treatment groups were then subdivided into the following four families of psychological intervention: (a) trauma-focused cognitive–behavioural therapy (TF-CBT, e.g. prolonged exposure, cognitive processing therapy); (b) eye movement desensitisation and reprocessing (EMDR); (c) other psychological interventions (e.g. psychoanalytic therapy, spiritual hypnosis-assisted treatment); and (d) multidisciplinary treatments (e.g. intensive multimodal group programmes, risk reduction through family therapy, teaching recovery techniques). The control groups were divided into passive control conditions (e.g. waiting lists, no treatment) and active control conditions (e.g. treatment as usual, supportive counselling). See Supplementary Appendix C for an overview of categorisations.

### Main outcome data

The main outcome concerned the short-term and long-term efficacy of treatments in terms of lowering PTSD symptomatology when compared with a given control condition (e.g. passive control conditions). Thus, we operationalised efficacy as the standardised mean difference in PTSD symptom severity (i.e. Hedges’ *g*; further detail is given in the ‘Statistical analysis’ section below) between comparison groups after treatment end-point (post-treatment) or at two follow-up assessments (see below). Consequently, the means, standard deviations and group sizes were extracted per assessment time point to calculate Hedges’ *g* for all relevant comparisons. We also aimed to analyse complex PTSD as a secondary treatment outcome. The follow-up periods were divided into follow-up 1 (FU1) and follow-up 2 (FU2). Assessments conducted up to 5 months after the end of treatment were categorised into FU1 and if multiple assessments fell into this category the closest one to 5 months was chosen. Assessments conducted more than 5 months after treatment end were categorised into FU2 and if multiple assessments fell into this category the longest reported follow-up was chosen. When both interview-based (i.e. clinician-administered) and self-report-based outcome data were reported, the former were prioritised over the latter.

### Coding of trial characteristics

The following trial characteristics were extracted (when reported): the number and length of treatment sessions, the intervention format (i.e. individual versus group and whether or not parents were involved in the treatment), the trauma type(s) that the sample had survived, the percentage of participants who reported one traumatic life event, the percentage of participants who reported more than one traumatic life event, the mean number of trauma exposures (irrespective of trauma type) across the sample, the country in which the trial was conducted, the baseline PTSD rate and the PTSD measure used to assess PTSD at baseline, the proportion of female participants, the length (in months) of the longest follow-up assessment, the mean age of the (total) sample or age range of the (total) sample if the mean was not reported, and whether results from ITT analyses or completer analyses were reported.

### Statistical analysis

Efficacy was estimated via Hedges’ *g* effect size. Hedges’ *g* was calculated by subtracting the group mean PTSD symptom severity score for the control condition from the group mean PTSD symptom severity score for the experimental condition at the given assessment time point (e.g. treatment end-point), dividing the difference by the pooled standard deviation and then multiplying the quotient by the sample size correction factor *J* = 1 – (3/(4 d.f. – 1)).^15^ Hedges’ *g* can conservatively be interpreted with Cohen's convention of small (0.2), medium (0.5) and large (0.8) effects.^16^

Random-effects meta-analyses were conducted using the metafor package (version 3.4-0) in R version 4.1.1 for Windows.^17,18^ Random-effects models were conducted (rather than fixed-effects models) in the light of expectably large heterogeneity in outcomes, given the broad focus of our research (i.e. various psychological interventions and populations). Factorial meta-analyses were carried out for the dichotomous definition of trauma frequency and meta-regressions were carried out for the continuous definition. However, analyses (including subgroup analyses) were only conducted when at least four independent trials had accumulated for the given comparison (*k* ≥ 4 for main analyses, *k* ≥ 4 per group for the dichotomous moderator analyses,^14^ and *k* ≥ 10 for the continuous moderator analyses, as recommended).^19^ To examine heterogeneity in outcomes, we calculated the *Q*-statistic, including its statistical significance, as well as the *I^2^*-statistic. *I^2^* indicates heterogeneity in outcomes in percentages. We further calculated prediction intervals, supplying an interval in which the true estimate is to be expected when similar future trials accumulate.^20,21^ We also calculated the numbers needed to treat (NNTs), which indicate the numbers of patients who need to be treated with the experimental condition compared with the control condition to achieve one additional treatment success.^22^ NNTs might be easier to interpret from a clinical perspective than standardised mean differences.

We performed various checks for detecting and addressing (potential) biases. We performed outlier-adjusted analyses whenever we detected one or more outliers. As recommended,^23^ we defined outliers as *g*-values that were extraordinarily high or low (i.e. scoring at least 3.3 standard deviations below or above the pooled *g* for the given comparison). We conducted additional moderator analyses to identify whether trial quality (see ‘Risk of bias assessment’ above) may bias results and potentially confound hypothesised effects. More specifically, we analysed in meta-regressions within the single- versus multiple-trauma categories whether or not trial quality was associated with outcomes when the evidence base was sufficiently large (*k* ≥ 10). As recommended,^19^ we only checked for potential publication bias when the evidence base was sufficiently large (*k* ≥ 10). We checked for potential publication bias using Egger's test of asymmetry.^24^ As recommended,^25^ we only performed the trim-and-fill method when the Egger's test was statistically significant. The trim-and-fill method supplies asymmetry-adjusted estimates by introducing hypothetical effects.

## Results

### Study synthesis

**Fig. 1:**
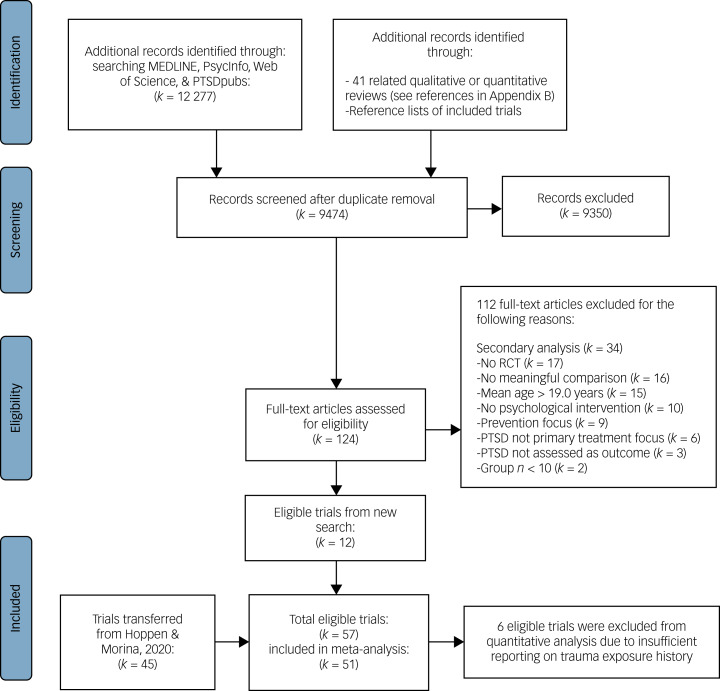
PRISMA flowchart of study selection.

Our database search yielded 9474 unique hits, including 41 related reviews that we also screened for eligible RCTs (see Supplementary Appendix B for their references). After the thorough title and abstract screening, 124 hits remained for full-text screening. After excluding articles that did not meet inclusion criteria, 12 new eligible RCTs remained, yielding 57 RCTs in total (i.e. 45 RCTs transferred from our previous search^2^). However, only 51 RCTs were included in at least one quantitative analysis; the other 6 trials were excluded owing to insufficient reporting on trauma exposure history. The PRISMA flowchart illustrates the study selection process (Fig. 1).

### Characteristics of included studies

All RCTs were reported in published journal articles, except one that was the subject of a doctoral dissertation.^26^ All trial reports were written in English (including the dissertation). The 57 RCTs involved data from 4295 children and adolescents, of whom 2305 were randomised to psychological interventions and 1990 to control conditions. The majority of trials (63.2%) utilised an interview-based outcome measure to assess PTSD outcomes. The (unweighted) mean duration of psychological treatments was 668.76 min, with large variation between trials (s.d. = 407.48). Just over one-third of trials (36.84%) involved parents in the treatment. Of the 57 trials, 48 reported in sufficient detail on the trauma exposure history of included participants to allow us to classify studies as single- versus multiple-trauma trials. Of those, the majority (70.8%, *k* = 34 trials) involved samples who had exclusively or mainly (i.e. ≥50%) survived multiple trauma rather than samples who had exclusively survived a single trauma (29.2%, *k* = 14). About half of the 57 eligible trials (52.6%, *k* = 30) did not report the mean number of trauma exposures. Although some of these trials reported on the mean number of experienced trauma types, we did not include these data in our analyses, to avoid blurring results. The analyses on trauma frequency as a continuous potential moderator of treatment efficacy was consequently based on the remaining 27 trials (47%) that sufficiently reported on the mean number of trauma exposures. Only six eligible trials reported neither information (i.e. no information regarding the dichotomous or the continuous definition of trauma exposure frequency). See Supplementary Appendix C, column 5, for an overview of the categorisation into single- versus multiple-trauma samples and the mean number of trauma exposures per sample, and the other columns for other trial characteristics of included trials more generally. The references of included trials are presented in Supplementary Appendix D. Although it was mentioned in several multiple-trauma trials that included patients presented with complex clinical presentations, none of the included trials assessed complex PTSD as a treatment outcome.

### Risk of bias assessment

The initial agreement rate between the independent raters (T.H.H. and N.M.) was good (91.3%). These authors discussed all discrepancies until agreement was reached. Study quality across trials was moderate on average, with an unweighted mean of 5.37 (s.d. = 1.64) (Supplementary Appendix E). The analyses regarding the potential influence of trial quality on the hypothesised effect of trauma frequency on treatment efficacy are described below.

### Intervention efficacy × trauma frequency (single versus multiple)

**Fig. 2:**
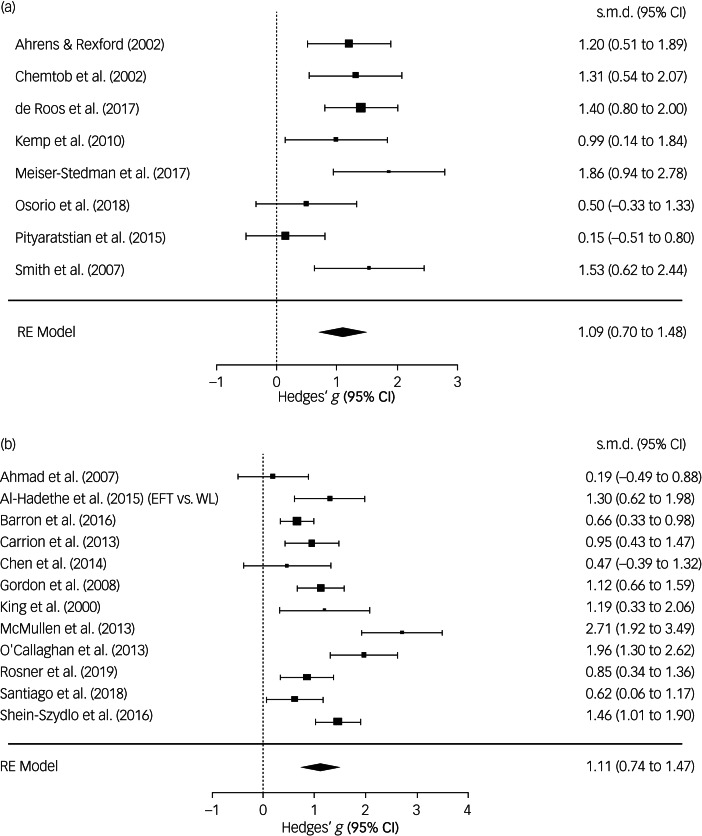
Forest plots depicting the efficacy of psychological interventions versus passive control conditions at treatment end-point in samples exposed to (a) a single trauma or (b) (mainly) multiple traumas. [EFT v. WL], the trial had more than two relevant arms: emotional freedom techniques versus waiting-list control condition; RE model, random-effects model. Data are shown for the extracted (primary) comparison; other comparisons were neglected to avoid data dependencies. References for the cited trials are listed in Supplementary Appendix D.

There were no statistical outliers in any of the performed analyses. Results indicated that psychological interventions are effective in treating both individuals who survived a single trauma and individuals who survived multiple traumas. Yet, in none of our analyses did we find evidence for differences in efficacy × trauma frequency. Three analyses on short-term efficacy were feasible in terms of the number of accumulated trials (Table 1). Psychological interventions as compared with passive control conditions at treatment end-point (i.e. short-term efficacy) were highly effective in lowering PTSD symptomatology in both samples who had survived single trauma (*g* = 1.09, 95% CI 0.70–1.48, *k* = 8) and samples who mainly or exclusively had survived multiple traumas (*g* = 1.11, 95% CI 0.74–1.47, *k* = 12), with no statistically significant difference in efficacy between the two groups (*P* = 0.978; Fig. 2).

**Table 1:** Post-treatment efficacy of psychological interventions for paediatric post-traumatic stress disorder for single- versus multiple-trauma trials

Comparison	Single versus multiple (trauma) exposures	Number of trials, *k*	Hedges’ *g*	95% CI (prediction interval)	*I*²	NNT	Moderation test, *P*
All interventions versus passive control conditions	Single	8	1.09***	**0.70 to 1.48 (0.20 to 1.98)**	52.88*	1.79	0.978
	Multiple	12	1.11***	0.74 to 1.47 (−0.04 to 2.26)	79.59***	1.77	
All interventions versus active control conditions	Single	1	n.a.	n.a.	n.a.	n.a.	n.a.
	Multiple	14	0.61***	0.34 to 0.89 (−0.28 to 1.51)	76.04***	2.98	
TF-CBT versus passive control conditions	Single	5	1.10***	0.54 to 1.66 (−0.06 to 2.26)	65.99*	1.78	0.598
	Multiple	7	1.33***	0.80 to 1.86 (−0.05 to 2.71)	84.66	1.53	
TF-CBT versus active control conditions	Single	1	n.a.	n.a.	n.a.	n.a.	n.a.
	Multiple	9	0.73***	0.40 to 1.06 (−0.16 to 1.63)	78.93***	2.53	
EMDR versus passive control conditions	Single	4	**1**.11***	**0.72 to 1.50 (0.65 to 1.58)**	9.99	1.76	n.a.
	Multiple	1	n.a.	n.a.	n.a.	n.a.	n.a.
TF-CBT versus other psychological interventions (EMDR, MDTs)	Single	4	0.03	−0.28 to 0.33 (−0.39 to 0.45)	22.38	63.53	0.796
	Multiple	9	0.15	−0.28 to 0.58 (−1.09 to 1.40)	84.90***	11.53	

NNT, number needed to treat; n.a., not applicable (i.e. number of trials too small (*k* < 4) to conduct analysis); TF-CBT, trauma-focused cognitive–behavioural therapy; EMDR, eye movement desensitisation and reprocessing; MDTs, multidisciplinary treatments (i.e. involving a mixture of techniques from at least two families, such as TF-CBT techniques + EMDR techniques). **Bold** indicates that both the 95% CI and the prediction interval exclude the null, highlighting large certainty in the respective efficacy.

*P < 0.05, ***P < 0.001.

The results in a subanalysis solely focusing on TF-CBT (rather than across various psychological interventions) in comparison with passive control conditions at treatment end-point yielded very similar results, with high efficacy in both single- and multiple-trauma trials (*g* ≥ 1.10), with no statistical evidence for differences in *g*-values (*P* = 0.598; Supplementary Appendix F). Similarly, we found that the short-term treatment efficacy of TF-CBT was similar to that of other psychological interventions (e.g. EMDR, multidisciplinary treatments) in both single- and multiple-trauma samples (Supplementary Appendix G and Supplementary Table 1).

Sensitivity analyses with the more conservative definition of multiple-exposure trials (i.e. ≥90% of sample with ≥2 lifetime trauma exposures) yielded very similar results. That is, psychological interventions were found effective for both single-eventtrauma-related PTSD and multiple-event-trauma-related PTSD for all comparisons at post-treatment, with no significant differences in efficacy in any of the performed analyses (Supplementary Appendix H).

Notably, the general evidence base on long-term outcome data remains relatively sparse, precluding all planned analyses. Most evidence concerning the long-term efficacy of psychological interventions is based on samples who mainly survived multiple (rather than single) traumas. For these multiple-trauma studies, there was evidence of an effect in the earlier follow-up window (<5 months) for passive control trials (*g* = 0.67) and in the longer follow-up window for active control trials (*g* = 0.49); there was no evidence of an effect for active control trials in the earlier follow-up window (*g* = 0.28). When restricting the data to trials evaluating TF-CBT, the effect remained for the passive control trials (earlier follow-up window: *g* = 0.45) and active control trials (in the longer follow-up window, *g* = 0.49) (Supplementary Appendix I).

Although we aimed to analyse trial quality as a potential confounding variable in the single- and multiple-trauma categories, meta-regressions were only feasible (*k* ≥ 10) for comparisons related to the multiple-trauma category and at treatment end-point. In these, trial quality was not associated with efficacy outcomes (Supplementary Appendix J).

### Intervention efficacy × trauma frequency (continuous)

In line with the results on trauma frequency defined dichotomously (i.e. single versus multiple), the analysis on trauma frequency as a continuous predictor of outcomes was not statistically significant. For the comparison of psychological interventions with passive control conditions at treatment end-point, the mean number of traumatic exposures was not significantly associated with treatment outcomes (*k* = 17; *b* = 0.02; *I*² = 76.51, *P* = 0.515). The analysis on psychological interventions versus active control conditions was precluded because of an insufficient number of available trials (*k* = 3), as were analyses on follow-up outcomes more generally.

## Discussion

The present work considered whether the efficacy of psychological interventions for PTSD in children and adolescents – already established in previous meta-analyses^2,3^ – differs depending on whether patients reported one or more lifetime trauma exposures. We hypothesised that the efficacy would be reduced (versus increased) for multiple (versus single) trauma exposures. The current evidence base of RCTs does not support this hypothesis. Rather, the data suggested that psychological interventions are largely effective in treating both individuals who have suffered single as well as individuals who have suffered multiple trauma exposures. Notably, comparison between single- and multiple-trauma trials, and analysis of the relationship between number of trauma exposures and efficacy, was only possible for trials that used passive control conditions. For such trials, both single- and multiple-trauma studies yielded large effects relative to controls in both the main analysis and the sensitivity analysis when we used a more conservative definition of multiple-exposure trials.

### Implications for clinical practice, training and treatment

We believe that these findings speak to critical issues regarding the provision of psychological therapies for children and adolescents with PTSD. First, multiple trauma is associated with a worse PTSD presentation.^4–6^ The finding that this particularly vulnerable population nevertheless responds well to intervention is encouraging and important for clinical practice and service planning. There are strong grounds for optimism that child and adolescent community mental health services can effectively treat PTSD in most of their patients even in the context of multiple trauma exposure (such as abuse or maltreatment), which may be the more typical PTSD presentation referred to such services. Second, this finding speaks to therapist training. A host of factors may act as barriers to the delivery of evidence-based interventions to individuals with PTSD, with therapist factors such as fears regarding delivering trauma-focused therapy and lack of training being widely reported.^27^ Recent studies^28–30^ point to increasing therapist reluctance to utilise evidence-based therapies with children exposed to multiple trauma. These findings may be used in training to highlight the body of knowledge that supports the efficacy of psychological therapies – with by far most accumulated evidence for TF-CBT – for youth who have experienced multiple traumas (including sexual violence and war). To increase the provision of evidence-based therapies to children and adolescents exposed to multiple traumas, therapists may need training not only in the interventions, but also in preparing and guiding inexperienced therapists in the process of clinical decision-making in complex situations with increased levels of general adversity, comorbidity and low levels of social support. Third, these findings may also have some implications for the treatment of complex PTSD (CPTSD), a disorder that may develop following prolonged and repeated traumatic events, in particular chronic traumatisation during childhood.^31^ Research into this disorder is still in its infancy, but the finding that multiple-trauma PTSD responds well to psychological therapies, in particular TF-CBT, suggests that the use of such interventions for children and adolescents with CPTSD should not be ruled out. Indeed, a single-arm pilot trial suggests that CPTSD does respond well to TF-CBT.^32^ Notably, the present results are in line with meta-analyses from the field of PTSD in adulthood.^8,33^ That is, psychological interventions, and TF-CBT in particular, are effective in the treatment of adult PTSD following complex traumatisation, including childhood trauma-related PTSD in adulthood.^8^

Although comparisons between trials that used active control arms were not possible, the effect size for trials that used an active control arm might be classed as medium (*g* = 0.61 at post-treatment). Though weaker than the effect for either single- or multiple-trauma treatment trials that used passive control conditions (as one might expect), this effect remains clinically significant and demonstrates the utility of trauma-focused psychological therapies over non-specific therapies. Non-specific approaches might be perceived by some as more appropriate for children and adolescents with more complex trauma histories, but trauma-focused psychotherapies yield superior outcomes compared with active control treatments and should be considered a first-line treatment for this population.

### Implications for future research

With respect to future research, the present review underlines some key points. First, future research might be helpfully directed towards improving the design quality of single-event PTSD trials; only one trial in our review used an active control condition. This makes it hard to establish how specific elements (e.g. the processing of traumatic memories through techniques such as producing a written narrative and imaginal reliving, or the use of cognitive restructuring) of trauma-focused psychological therapies are integral to treatment response for single-event trauma, over and above non-specific effects (e.g. therapist attention, natural recovery, regression to the mean). Second, the general overall quality of trials was modest. Although study quality was not related to the efficacy of PTSD treatment following multiple-trauma exposure, it is clear that much can be done to improve the quality of evidence in this area. In particular, many trials were small (<50 participants) and 40% did not use an ITT approach in their analysis. Less than a quarter required participants to meet full diagnostic criteria for PTSD; although this may reflect concerns regarding the utility and appropriateness of diagnostic tools in this age group, it may mean that the evidence base pertains to milder cases. Third, future trials urgently need to include longer-term follow-up assessments. A significant issue for the single-event PTSD evidence base is that existing RCTs frequently used a waiting-list design, where follow-up comparisons are unethical (i.e. denying much-needed treatment to the waiting-list control condition for a long period).

### Strengths and limitations

The present review has several strengths, in particular the close attention to RCT control arm type and study quality, the inclusion of post-treatment as well as follow-up findings and the use of two ways of exploring the effect of trauma frequency on treatment efficacy. Moreover, the present work was conducted in accordance with gold standard guidelines on meta-analysis (i.e. PRISMA guidelines), all steps were carried out by at least two researchers working independently and discrepancies were solved through through discussions until consensus was reached. Last, we adjusted for the fact that dichotomising a continuous variable is always associated with a certain degree of arbitrariness and might blur results. That is, we assessed the potential influence of trauma frequency on efficacy outcomes both dichotomously as well as continuously and results were in line.

Some limitations also need to be noted. First, some studies had to be excluded from the quantitative analyses as they did not report trauma frequency. Therefore, we strongly encourage authors to report on this important clinical variable. Second, our definition of multiple trauma can be criticised. Our cut-off for being considered a multiple-exposure trial was that at least 50% of participants had suffered multiple trauma exposures. However, we carried out a sensitivity analysis with a more conservative definition of multiple-exposure trial (i.e. at least 90% of participants reporting multiple lifetime trauma exposures) and results and conclusions were very similar (i.e. no differential treatment efficacy between single- versus multiple-exposure trials). Third, it would have been desirable also to focus on other metrics of treatment success beyond standardised mean differences. However, in the field of paediatric PTSD there is no gold standard definition of treatment response or clinically meaningful change. Applied definitions of treatment success varied substantially between included trials (e.g. a decrease in symptom severity of at least 50% pre- to post-treatment, participant not scoring within the clinical range on a self-report measure at post-treatment, participant not meeting diagnostic criteria in a clinical interview at post-treatment) and most trials (and particularly older trials) reported only group means. Fourth, some of the included trials were of low methodological quality. Although trial quality across all trials was moderate (mean 5.37 out of 8), some trials had a low quality sum score (e.g. owing to *n* ≤ 50 or application of completer analyses). However, all included trials fulfilled our rather strict inclusion criteria and thus were of sufficient methodological rigour to warrant valid analyses. For instance, we only included trials with *n* ≥ 20, in an effort to exclude chance findings. Last, the generalisability of results from RCTs to clinical practice might be impeded by the (required) standardisation (e.g. inclusion and exclusion criteria for participants, highly standardised treatments).

## Data Availability

All presented data are publicly accessible. The datasets and R scripts to reproduce results are available from the corresponding author on request.

## Supplementary material

For supplementary material accompanying this paper visit https://doi.org/10.1192/bjp.2023.24. click here to view supplementary material
