# Supplementary material for: Efficacy of psychological interventions for post-traumatic stress disorder in children and adolescents exposed to single versus multiple traumas: meta-analysis of randomised controlled trials
Source: Br J Psychiatry. 2023 May;222(5):196–203. doi: 10.1192/bjp.2023.24 (PMC10895514; doi:10.1192/bjp.2023.24)
Supplement: Supplementary file 1 [file S0007125023000247sup001.docx]

**Supplementary material to the manuscript: The efficacy of psychological interventions for PTSD in children and adolescents exposed to single vs. multiple traumas.
Meta-analysis of randomized controlled trials**

**Appendix A. Search strategy**

**Appendix B. References of screened related review articles**

**Appendix C. Characteristics of included trials**

**Appendix D. References of included trials**

**Appendix E. Trial quality of included trials**

**Appendix F. Forest plots depicting the efficacy of trauma-focused cognitive behavior therapy vs. passive control conditions at treatment endpoint in samples exposed to a single trauma (top) or multiple traumas (bottom)**

**Appendix G. Forest plots depicting the efficacy of trauma-focused cognitive behavior therapy vs. other psychological interventions at treatment endpoint in samples exposed to a single trauma (top) or multiple traumas (bottom)**

**Appendix H. Sensitivity analysis^1^: Efficacy of psychological interventions for pediatric PTSD for single vs. multiple trauma trials at posttreatment**

**Appendix I. Long-term efficacy of psychological interventions for pediatric PTSD in samples exposed to a single trauma vs. multiple traumas**

**Appendix J.** **Trial quality as a potential moderator of short-term efficacy of psychological interventions for pediatric PTSD in samples exposed to a single trauma vs. multiple traumas**

**Appendix A. Search strategy**

| Databases | Search Terms |
| --- | --- |
| MEDLINE and PsycINFO | ( TI ( ptsd OR ptss OR post-traumatic stress OR posttraumatic stress OR post-traumatic syndrome OR posttraumatic syndrome) OR AB ( ptsd OR ptss OR post-traumatic stress OR posttraumatic stress OR post-traumatic syndrome OR posttraumatic syndrome ) OR SU ( ptsd OR ptss OR post-traumatic stress OR posttraumatic stress OR post-traumatic syndrome OR posttraumatic syndrome ) ) AND ( TI ( treatment* OR intervention* OR therap* OR psychotherap* OR exposure OR counse*ing OR trial* ) OR AB ( treatment* OR intervention* OR therap* OR psychotherap* OR exposure OR counse*ing OR trial* ) OR SU ( treatment* OR intervention* OR therap* OR psychotherap* OR exposure OR counse*ing OR trial* ) ) |
| PTSDpubs | (ptsd OR ptss OR post-traumatic stress OR posttraumatic stress OR post-traumatic syndrome OR posttraumatic syndrome) AND (treatment* OR intervention* OR therap* OR psychotherap* OR exposure OR counse*ing OR trial*) |
| Web of Science | ALL=( ptsd OR ptss OR post-traumatic stress OR posttraumatic stress OR post-traumatic syndrome OR posttraumatic syndrome ) AND ALL=( treatment* OR intervention* OR therap* OR psychotherap* OR exposure OR counse*ing OR trial* ) |
| Note that the search string contains APA thesaurus/MeSH search terms (i.e., “posttraumatic stress”, “treatment”, “intervention”, “psychotherapy”, “exposure” and “counseling” as well as additional terms (e.g., “post-traumatic stress”, “PTSD”, “trial”, “therapy”) in case a particular trial was not registered under these APA thesaurus/MeSH search terms. | |

**Appendix B.** **References of screened related review articles**

Al-Tamimi, S. A. G. A., & Leavey, G. [G.] (2021). Community-based interventions for the treatment and management of conflict-related trauma in low-middle income, conflict-affected countries: A realist review. *Journal of Child & Adolescent Trauma*, *15*(2), 441–450.

Astill Wright, L., Horstmann, L., Holmes, E. A., & Bisson, J. I. (2021). Consolidation/reconsolidation therapies for the prevention and treatment of PTSD and re-experiencing: A systematic review and meta-analysis. *Translational Psychiatry*, *11*(1), 453.

Baetz, C. L., Branson, C. E., Weinberger, E., Rose, R. E., Petkova, E., Horwitz, S. M., & Hoagwood, K. E. (2022). The effectiveness of PTSD treatment for adolescents in the juvenile justice system: A systematic review. *Psychological Trauma: Theory, Research, Practice, and Policy*, *14*(4), 642–652.

Bamford, J., Fletcher, M., & Leavey, G. [Gerard] (2021). Mental health outcomes of unaccompanied refugee minors: A rapid review of recent research. *Current Psychiatry Reports*, *23*(8), 46.

Bennett, R. S., Denne, M., McGuire, R., & Hiller, R. M. (2021). A systematic review of controlled-trials for PTSD in maltreated children and adolescents. *Child Maltreatment*, *26*(3), 325–343.

Braito, I., Rudd, T., Buyuktaskin, D., Ahmed, M., Glancy, C., & Mulligan, A. (2021). Review: Systematic review of effectiveness of art psychotherapy in children with mental health disorders. *Irish Journal of Medical Science (1971-)*, *191*, 1369–1383.

Correll, C. U., Cortese, S., Croatto, G., Monaco, F., Krinitski, D., Arrondo, G., . . . Estradé, A. (2021). Efficacy and acceptability of pharmacological, psychosocial, and brain stimulation interventions in children and adolescents with mental disorders: An umbrella review. *World Psychiatry*, *20*(2), 244–275.

Danzi, B. A., & La Greca, A. M. (2021). Treating children and adolescents with posttraumatic stress disorder: Moderators of treatment response. *Journal of Clinical Child & Adolescent Psychology*, *50*(4), 510–516.

Darawshy, N. A.‑S., Gewirtz, A., & Marsalis, S. (2020). Psychological intervention and prevention programs for child and adolescent exposure to community violence: A systematic review. *Clinical Child and Family Psychology Review*, *23*(3), 365–378.

Ford, J. D. (2021). Progress and limitations in the treatment of complex PTSD and developmental trauma disorder. *Current Treatment Options in Psychiatry*, *8*(1), 1–17.

Galvan, M. S., Lueke, A. E., Mansfield, L.‑T. E., & Smith, C. A. (2021). A systematic research review: How to best treat post-traumatic stress disorder in children post-natural disaster. *Journal of Human Behavior in the Social Environment*, *31*(6), 701–715.

Gilbert, R., Abel, M. R., Vernberg, E. M., & Jacobs, A. K. (2021). The use of psychological first aid in children exposed to mass trauma. *Current Psychiatry Reports*, *23*(9).

Gómez, G., Basagoitia, A., Burrone, M. S., Rivas, M., Solís-Soto, M. T., Dy Juanco, S., & Alley, H. (2021). Child-focused mental health interventions for disasters recovery: A rapid review of experiences to inform return-to-school strategies after COVID-19. *Frontiers in Psychiatry*, *12*.

Hediger, K., Wagner, J., Künzi, P., Haefeli, A., Theis, F., Grob, C., . . . Gerger, H. (2021). Effectiveness of animal-assisted interventions for children and adults with post-traumatic stress disorder symptoms: A systematic review and meta-analysis. *European Journal of Psychotraumatology*, *12*(1), 1879713.

Hoogsteder, L. M., Thije, L. ten, Schippers, E. E., & Stams, G. J. J. (2022). A meta-analysis of the effectiveness of EMDR and TF-CBT in reducing trauma symptoms and externalizing behavior problems in adolescents. *International Journal of Offender Therapy and Comparative Criminology*, *66*(6-7), 735–757.

Huang, T., Li, H., Tan, S., Xie, S., Cheng, Q., Xiang, Y., & Zhou, X. (2022). The efficacy and acceptability of exposure therapy for the treatment of post-traumatic stress disorder in children and adolescents: A systematic review and meta-analysis. *BMC Psychiatry*, *22*.

John-Baptiste Bastien, R., Jongsma, H. E., Kabadayi, M., & Billings, J. (2020). The effectiveness of psychological interventions for post-traumatic stress disorder in children, adolescents and young adults: A systematic review and meta-analysis. *Psychological Medicine*, *50*(10), 1598–1612.

Kaptan, S. K., Dursun, B. O., Knowles, M., Husain, N., & Varese, F. (2021). Group eye movement desensitization and reprocessing interventions in adults and children: A systematic review of randomized and nonrandomized trials. *Clinical Psychology & Psychotherapy*, *28*(4), 784–806.

Kletter, H., Matlow, R., Tanovic, S., & Carrion, V. (2021). Cue-centered therapy for youth experiencing posttraumatic symptoms. *Current Treatment Options in Psychiatry*, *8*(3), 125–140.

Lawton, K., & Spencer, A. (2021). A full systematic review on the effects of cognitive behavioural therapy for mental health symptoms in child refugees. *Journal of Immigrant and Minority Health*, *23*(3), 624–639.

Le Roux, I. H., & Cobham, V. E. (2021). Psychological interventions for children experiencing PTSD after exposure to a natural disaster: A scoping review. *Clinical Child and Family Psychology Review*, *25*, 249–282.

Manzoni, M., Fernandez, I., Bertella, S., Tizzoni, F., Gazzola, E., Molteni, M., & Nobile, M. (2021). Eye movement desensitization and reprocessing: The state of the art of efficacy in children and adolescent with post traumatic stress disorder. *Journal of Affective Disorders*, *282*, 340–347.

Mavranezouli, I., Megnin‐Viggars, O., Daly, C., Dias, S., Stockton, S., Meiser‐Stedman, R., . . . Pilling, S. (2020). Research review: Psychological and psychosocial treatments for children and young people with post‐traumatic stress disorder: A network meta‐analysis. *Journal of Child Psychology and Psychiatry*, *61*(1), 18–29.

McGuire, A., Steele, R. G., & Singh, M. N. (2021). Systematic review on the application of trauma-focused cognitive behavioral therapy (TF-CBT) for preschool-aged children. *Clinical Child and Family Psychology Review*, *24*(1), 20–37.

McTavish, J. R., Santesso, N., Amin, A., Reijnders, M., Ali, M. U., Fitzpatrick-Lewis, D., & MacMillan, H. L. (2021). Psychosocial interventions for responding to child sexual abuse: A systematic review. *Child Abuse & Neglect*, *116*(1), 104203.

McWey, L. M. (2022). Systemic interventions for traumatic event exposure: A 2010–2019 decade review. *Journal of Marital and Family Therapy*, *48*(1), 204–230.

Noll, J. G. (2021). Child sexual abuse as a unique risk factor for the development of psychopathology: The compounded convergence of mechanisms. *Annual Review of Clinical Psychology*, *17*, 439–464.

Peters, W., Rice, S., Alvarez‐Jimenez, M., Hetrick, S. E., Halpin, E., Kamitsis, I., . . . Bendall, S. (2022). Relative efficacy of psychological interventions following interpersonal trauma on anxiety, depression, substance use, and PTSD symptoms in young people: A meta‐analysis. *Early Intervention in Psychiatry*, *16*(11), 1175–1184.

Pfefferbaum, B., Nitiéma, P., & Newman, E. (2021). A critical review of effective child mass trauma interventions: What we know and do not know from the evidence. *Behavioral Sciences*, *11*(2), 25–39.

Plaisted, H., Waite, P., Gordon, K., & Creswell, C. (2021). Optimising exposure for children and adolescents with anxiety, OCD and PTSD: A systematic review. *Clinical Child and Family Psychology Review*, *24*(2), 348–369.

Ramírez-Guarín, V., Codina, N., & Pestana, J. V. (in print). A systematic review of psychosocial interventions for child soldiers: Types, length and main findings. *Journal of Social Work Practice*.

Siehl, S., Robjant, K., & Crombach, A. (2021). Systematic review and meta-analyses of the long-term efficacy of narrative exposure therapy for adults, children and perpetrators. *Psychotherapy Research*, *31*(6), 695–710.

Simmons, C., Meiser-Stedman, R., Baily, H., & Beazley, P. (2021). A meta-analysis of dropout from evidence-based psychological treatment for post-traumatic stress disorder (PTSD) in children and young people. *European Journal of Psychotraumatology*, *12*(1), 1947570.

Thomas, F. C., Puente‐Duran, S., Mutschler, C., & Monson, C. M. (2022). Trauma‐focused cognitive behavioral therapy for children and youth in low and middle‐income countries: A systematic review. *Child and Adolescent Mental Health*, *27*(2), 146–160.

Uppendahl, J. R., Alozkan-Sever, C., Cuijpers, P., Vries, R. de, & Sijbrandij, M. (2020). Psychological and psychosocial interventions for PTSD, depression and anxiety among children and adolescents in low-and middle-income countries: A meta-analysis. *Frontiers in Psychiatry*, *10*, 933.

Wergeland, G. J. H., Riise, E. N., & Öst, L.‑G. (2021). Cognitive behavior therapy for internalizing disorders in children and adolescents in routine clinical care: A systematic review and meta-analysis. *Clinical Psychology Review*, *83*, 101918.

Xiang, Y., Cipriani, A., Teng, T., Del Giovane, C., Zhang, Y., Weisz, J. R., . . . Barth, J. (2021). Comparative efficacy and acceptability of psychotherapies for post-traumatic stress disorder in children and adolescents: A systematic review and network meta-analysis. *Evidence-Based Mental Health*, *24*(4), 153–160.

Yohannan, J., Carlson, J. S., & Volker, M. A. (2022). Cognitive behavioral treatments for children and adolescents exposed to traumatic events: A meta‐analysis examining variables moderating treatment outcomes. *Journal of Traumatic Stress*, *35*(2), 706–717.

Young, G. (2022). Psychotherapeutic Change Mechanisms and Causal Psychotherapy: Applications to Child Abuse and Trauma. *Journal of Child & Adolescent Trauma*, *15*, 911–923.

Zhang, S., Conner, A., Lim, Y., & Lefmann, T. (2021). Trauma-informed care for children involved with the child welfare system: A meta-analysis. *Child Abuse & Neglect*, *122*, 105296.

**Appendix C. Characteristics of included trials**

| **Publication,**  **conditions &**  **(number of sessions)** | n | Intervention  (format of delivery, parent involvement) | Trauma type | Trauma exposure freq.: single vs. multiple (mean nr of exposure) | Country | Basline PTSD rate in % (measure) | % female | outcome measure (PTSD) | Longest FU | Age, mean or range (SD) | Analysis | Quality sum score (out of 8) |
| --- | --- | --- | --- | --- | --- | --- | --- | --- | --- | --- | --- | --- |
| Ahmad et al. (2007)  EMDR (8 sessions, 45 min.)  WL | 17  16 | EMDR  (individual) | Multiple types | Multiple (n.a.) | Sweden | 100 (DICA) | 60.61 | PTSS-C | n.a. | 6–16  (10.0) | ITT | 4 |
| Ahrens & Rexford (2002)  TF-CBT (8 sessions, 60 min.)  WL | 19  19 | CPT  (group) | Physical violence | Single (1) | USA | 100 (PSS-SR) | 0 | PSS-SR | n.a. | 15–18  (16.4) | Compl. | 3 |
| Al-Hadethe et al. (2015)  TF-CBT  Other  WL | 19  20  20 | NET (individual)  EFT (individual) | War | Multiple* (n.r.) | Iraq | 100 (SPTSS) | 0 | SPTSS | 12 | 16-19 | Compl. | 2 |
| Auslander et al. (2017)  TF-CBT (10 sessions, 90 min.)  TAU (n.r.) | 15  10 | GAIN  (group, parent involvement) | Multiple types (child welfare) | Multiple* (n.a.) | USA | 67 (CPSS) | 100 | CPSS | r.b.i. | 12-18  (14.7) | Compl. | 5 |
| Auslander et al. (2020)  TF-CBT (14 sessions, 90 min)  UC (n.r., n.r.) | 115  108 | CBITS (group, individual, parent involvement) | Multiple types (child welfare) | Multiple* (n.a.) | USA | 54 (CPSS) | 100 | CPSS | 3 | 12-19  (14.89) | ITT | 6 |
| Barron et al. (2016)  TF-CBT (5 sessions, 60 min.)  WL | 79  75 | TRT  (group) | War | Multiple* (18.85) | Palestine | n.r. | 59.71 | CRIES | n.a. | 11-18  (13.5) | ITT | 7 |
| Barron et al. (2020)  TF-CBT (5 sessions, 90 min.) WL | 14  16 | TRT  (group) | Drug violence | Multiple (n.a.) | Brazil | 96.67 (CRIES) | 46.67 | CRIES | n.a. | 8-13  (10.1) | ITT | 3 |
| Carrion et al. (2013)  MDT (15 sessions, 50 min.)  WL | 38  27 | Cue-centered treatment (individual, parent involvement) | Physical violence | Multiple* (5.03) | USA | n.r. | 40.00 | UCLA PTSD-RI | n.a. | 8–17  (11.6) | ITT | 7 |
| Catani et al. (2009)  TF-CBT (6 sessions, 60-90 min.)  Meditation (6 sessions, 60-90 min.) | 16  15 | KIDNET  (individual) | Multiple types | Multiple* (n.a.) | Sri Lanka | n.a.^a^ (UPID) | 45.16 | UPID | 6 | 8–14  (12.0) | ITT | 6 |
| Celano et al. (1996)  TF-CBT (8 sessions, 60 min.)  TAU (8 sessions, 60 min.) | 15  17 | Recovering from abuse program  (individual, parent involvement in  both conditions) | Sexual assault | Multiple* (n.a.) | USA | n.r. | 100 | CITES-R | n.a. | 8–13  (10.5) | Compl. | 4 |
| Chemtob et al. (2002)  EMDR (3 sessions, n.r.)  WL | 17  15 | EMDR  (individual) | Disaster | Single (1) | USA | 100 (CRI) | 68.75 | CRI | n.a. | 6–12  (8.4) | Compl. | 5 |
| Chen et al. (2004)  MDT (6 sessions, 60 min.)  Support (ACC)  WL |  | TRT+ (group), general support (individual) | Parental loss and earthquake | Multiple*  (n.r.) | China | n.r. | 68.00 | CRIES | 3 | 14.50 (0.71) | Compl. | 2 |
| Cohen et al. (2004)  TF-CBT (12 sessions, 45 min.)  CCT (12 sessions, 45 min.) | 89  91 | TF-CBT  CCT  (individual, parent involvement in  both conditions) | Sexual assault | Multiple* (4) | USA | 89 (K-SADS) | 78.82 | K-SADS | 12 | 8–14  (10.7) | Compl. | 5 |
| Cohen, et al. (2005)  TF-CBT (12 sessions, 45 min.)  NST (12 sessions, 45 min.) | 41  41 | TF-CBT  (individual, parent involvement in both conditions) | Sexual assault | Multiple (n.a.) | USA | n.r. | 68.29 | TSCC | 12 | 8–15  (11.4) | ITT | 6 |
| Cohen et al. (2011)  TF-CBT (8 sessions, 45 min.)  CCT (8 sessions, 45 min.) | 64  60 | TF-CBT  CCT  (individual, parent involvement in both conditions) | Multiple types | Multiple (n.a.) | USA | 25 (K-SADS) | 50.81 | K-SADS | n.a. | 7–14  (9.6) | ITT | 7 |
| Danielson et al. (2012)  MDT (23 sessions, 60-90 min.)  TAU (13 sessions, n.r.) | 15  15 | RRFT  (individual, parent involvement  in both conditions) | Sexual assault | Multiple (n.a.) | USA | n.r. | 88.00 | UPID | 6 | 13–17  (14.8) | ITT | 5 |
| Dawson et al. (2018)  TF-CBT (6 sessions, 60 min.)  Other (6 sessions, 60 min.) | 32  32 | TF-CBT vs. PST  (individual &  parent involvement  in both conditions) | War and Tsunami | Multiple (n.a.) | Indonesia | n.r. | 48.44 | UCLA PTSD-RI | 12 | 7-14  (10.7) | ITT | 5 |
| de Roos et al. (2011)  TF-CBT (4 sessions, 60 min.)  EMDR (4 sessions, 60 min.) | 21  19 | TF-CBT, EMDR (individual, parent involvement in both conditions) | Explosion | Single (1) | NL | 17.3 (UCLA PTSD-RI) | 44.23 | UCLA PTSD-RI | 3 | 4–18  (10.1) | ITT | 6 |
| de Roos et al. (2017)  EMDR (6 sessions, 45 min.)  TF-CBT (6 sessions, 45 min.)  WL | 43  42  18 | EMDR  CBWT  (individual) | Multiple  types | Single  (1) | NL | 61.2 (CRTI) | 57.28 | CRTI | 12  (no WL  FU) | 8-18  (13.06) | ITT | 7 |
| Deblinger et al. (1996)  TF-CBT-c (12 sessions, 45 min.)  TF-CBT-c&p (12 sessions 90 min.)  TF-CBT-p (12 sessions, 45 min.)  TAU (n.r.) | 24  22  22  22 | TF-CBT  (individual, child only, child & parent,  parent only) | Sexual assault | Multiple (n.a.) | USA | 71 (K-SADS) | 83.00 | K-SADS | 24 | 7–13  (9.8) | Compl. | 3 |
| Diehle et al. (2015)  TF-CBT (12 sessions, 60 min.)  EMDR (12 sessions, 60 min.) | 23  25 | TF-CBT  EMDR  (individual, parent involvement in both conditions) | Multiple types | Single (1) | NL | 33 (CAPS-CA) | 62.50 | CAPS-CA | n.a. | 8–18  (12.9) | ITT | 6 |
| Dorsey et al. (2020)  TF-CBT (15 sessions, n.r.)  UC (n.r., n.r.) | 96  96 | TF-CBT  UC  (individual & group) | Parental death | Multiple (5.5) | Kenya  /urban | 100 (CPSS) | 50 | CPSS | 12 | 7-13  (10.7) | ITT | 7 |
| Dorsey et al. (2020)  TF-CBT (15 sessions, n.r.)  UC (n.r., n.r.) | 64  64 | TF-CBT  UC  (individual & group) | Parental death | Multiple (4.5) | Kenya  /rural | 100 (CPSS) | 50 | CPSS | 12 | 7-13  (10.2) | ITT | 7 |
| Dorsey et al. (2020)  TF-CBT (15 sessions, n.r.)  UC (n.r., n.r.) | 64  64 | TF-CBT  UC  (individual & group) | Parental death | Multiple (3.3) | Tanzania/  rural | 100 (CPSS) | 50 | CPSS | 12 | 7-13  (10.5) | ITT | 7 |
| Ertl et al. (2011)  TF-CBT (8 sessions, 90-120 min.)  SC (8 sessions, 90-120 min.)  WL | 26^b^  24^b^  28^b^ | KIDNET (individual) | War | Multiple* (21.07) | Uganda | 100 (CAPS-CA) | 55.29 | CAPS-CA | 12 | 12-25 (18.0) | Compl. | 6 |
| Foa et al. (2013)  TF-CBT (14 sessions, 60-90 min.)  SC (14 sessions, 60-90 min.) | 31  30 | PE  (individual) | Sexual assault | n.r.  (n.r.) | USA | 100 (CPSS-I) | 100 | CPSS-I | 12 | 13–18  (15.3) | ITT | 7 |
| Ford et al. (2012)  MDT (12 sessions, 50 min.)  TAU (12 sessions, 50 min.) | 33  26 | TARGET  (individual) | Multiple types | Multiple* (n.a.) | USA | 63 (CAPS-CA) | 100 | CAPS-CA | n.a. | 13–17  (14.7) | ITT | 6 |
| Gilboa-Schechtman et al. (2010)  TF-CBT (12–15 sessions, 60-90 min.)  PDP (15–18 sessions, 60-90 min.) | 19  19 | PE,  PDP  (individual) | Multiple types | Single (1) | Israel | 100 (CPSS) | 63.16 | CPSS | 17 | 12–18  (14.1) | ITT | 7 |
| Goldbeck et al. (2016)  TF-CBT (12 sessions, 90 min.)  WL | 76  83 | TF-CBT  (individual,  parent involvement) | Multiple  types | n.r. (6.35) | Germany | 75.5 (CAPS-CA) | 71.70 | CAPS-CA | n.a. | 7-17  (13.03) | ITT | 7 |
| Gordon et al. (2008)  MDT (12 sessions, 120 min.)  WL | 38  40 | Mind-body skills group  (group) | War | Multiple* (n.a.) | Kosovo | 100 (HTQ) | 75.61 | HTQ | n.a. | 14–18  (16.3) | Compl. | 6 |
| Hitchcock et al. (2021)  TF-CBT (12 sessions, n.r.) TAU (n.r., n.r.) | 18  19 | CBT-3M  (individual, parent involvement) | Multiple types | Single (1) | UK | 100 (DIPA, diagnosis PTSD-YC) | 51.35 | YCPC | r.b.i. | 3-8  (6.26) | ITT | 6 |
| Jensen et al. (2014)  TF-CBT (12-15 sessions, 45 min.)  TAU (n.r.) | 55  61 | TF-CBT  (individual, parent involvement in both  conditions) | Multiple types | Multiple (n.a.) | Norway | 66.7 (CAPS-CA) | 79.49 | CAPS-CA (post) CPSS (FUs) | 18 | 10–18  (15.1) | ITT | 7 |
| Kameoka et al. (2020)  TF-CBT (12 sessions, 95 min.)  WL | 14^b^  16^b^ | TF-CBT  (individual, parent involvement) | Multiple types | n.r. (3.1) | Japan | 100 (K-SADS) | 73.3 | K-SADS | 1 | 6-18  (13.9) | ITT | 6 |
| Kemp et al. (2010)  EMDR (4 sessions, 60 min.)  WL | 12  12 | EMDR  (individual) | Motor vehicle accident | Single (1) | Australia | n.r. | 44.44 | CPTS-RI | n.a. | 6–12  (8.9) | Compl. | 3 |
| King et al. (2000)  TF-CBT-c (20 sessions, 50 min.)  TF-CBT-c&p (20 sessions, 50 min.)  WL | 12  12  12 | TF-CBT  (individual, child only, child & parent involvement) | Sexual assault | Multiple* (7.64) | Australia | 69 (ADIS) | 69.44 | ADIS | 3 | 5–17  (11.5) | ITT | 4 |
| Langley et al. (2015)  MDT (10 group+2-3 individual sessions, 50-60 min. + 30-50 min.)  WL | 35  36 | Bounce Back  (individual  & group,  parent involvement) | Multiple  types | n.r. (4.7) | USA | n.r. | 50.00 | UPID | r.b.i. | 1st-5th  grade  (7.7) | Compl. | 6 |
| Lesmana et al. (2009)  Other (1 session, 30 min.)  WL | 48  178 | SHAT  (group) | Terrorist attack | Single  (1) | Indonesia | 100 (n.r.) | 52.70 | Self-developed measure based on DSM-IV-TR criteria | 24 | 9.83  (1.53) | ITT | 2 |
| McMullen et al. (2013)  TF-CBT (15 sessions, 45 min.)  WL | 24  24 | TF-CBT  (group) | War | Multiple* (14.35) | DR Congo | n.r. | 0 | UCLA PTSD-RI | n.a. | 13–17  (15.8) | Compl. | 5 |
| Meentken et al. (2020)  EMDR (3.5 sessions, 50 min.)  CAU (n.r, n.r.) | 37^b^  37^b^ | EMDR  (individual, parent involvement) | Multiple  types | Multiple (4.01) | NL | 100 (CAPS-CA) | 33.78 | CRTI | 2 | 4-15  (9.6) | ITT | 6 |
| Meiser-Stedman et al. (2017)  TF-CBT (10 sessions, 90 min.)  WL | 14  15 | CT  (individual) | Multiple types | Single (1) | UK | 75.9 (CPTSDI) | 72.41 | CPTSDI | n.a. | 8-17  (13.3) | ITT | 5 |
| Molero et al. (2019)  EMDR (9 sessions, first 95 min., subsequent sessions M = 48min.)  WL | 93  91 | EMDR-IGTP-OTS (group) | Multiple types | n.r.  (n.r.) | Spain | n.r. | 0 | PCL-5 | 3 | 13-17  (16.36) | Compl. | 5 |
| Murray et al. (2015)  TF-CBT (10-16 sessions, 60-90 min.)  TAU (NR) | 131  126 | TF-CBT (individual) | Multiple types | Multiple* (n.a.) | Zambia | n.r. | 50.97 | UPID | n.a. | 5-18  (13.6) | ITT | 7 |
| O'Callaghan et al. (2013)  TF-CBT (15 sessions, 45 min.)  WL | 24  28 | TF-CBT  (group, parent involvement) | War and sexual assault | Multiple* (12.1) | DR Congo | 60 (UCLA  PTSD-RI) | 100 | UCLA PTSD-RI | n.a. | 12–17  (16.1) | ITT | 7 |
| O'Callaghan et al. (2015)  TF-CBT (9 sessions, 90 min)  MDT (9 sessions, 90 min.)  (WL not used because  convenience sample) | 26  24 | TF-CBT,  eclectic psychosocial intervention  (group, parent involvement in  both active conditions) | War | Multiple* (19.7) | DR Congo | 92 (UCLA PTSD-RI) | 42.00 | UCLA PTSD-RI | 6 | 8–17  (14.8) | ITT | 7 |
| Osorio et al. (2018)  EMDR (6 sessions, first 106 min., subsequent sessions M=53 min.)  WL | 11  12 | EMDR-IGTP-OTS (group) | Cancer | Single (1) | Mexiko | n.r. | 43.48 | PCL-5 | 3 | 13-21  (16.71) | ITT | 6 |
| Peltonen & Kangaslampi (2019)  TF-CBT (7-10 sessions, 90 min.)  TAU (n.r., 45-90 min.) | 29  21 | NET (individual) | Multiple  types | Multiple* (n.a.) | Finland | n.r. | 42.00 | CRIES | r.b.i. | (13.24) | ITT | 4 |
| Pityaratstian et al. (2015)  TF-CBT (3 sessions, 120 min.)  WL | 18  18 | TF-CBT  (group) | Tsunami | Single (1) | Thailand | 100 (UCLA PTSD-RI) | 72.22 | UCLA PTSD-RI | 4 | 10-15  (12.3) | ITT | 4 |
| Roque-Lopez et al. (2021)  MDT (seven days)  TAU (seven days) | 22  22 | EMDR-IGTP-OTS (group, individual if required) | Multiple types | Multiple* (5.25) | Colombia | n.r. | 100 | SPRINT | 2 | 13-16  (14.05) | Compl. | 3 |
| Rosner et al. (2019)  TF-CBT (30 sessions, 50 min.)  WL | 44  44 | D-CPT (individual) | Sexual assault and physical violence | Multiple (n.a.) | Germany | 100 (CAPS-CA) | 85.23 | CAPS-CA | 3 | 14-21  (18.1) | ITT | 8 |
| Rossouw et al. (2020)  TF-CBT (7-14 sessions, 60 min.)  SC (7-14 sessions, 60 min.) | 31  32 | PE, SC (individual) | Multiple types | n.r.  (n.r.) | South Africa | 100 (MINI-KID) | 87.30 | CPSS-I | 6 | 13–18  (15.35) | ITT | 8 |
| Ruf et al. (2010)  TF-CBT (8 sessions, 90-120 min.)  WL | 13  13 | KIDNET  (individual) | War | Multple (4.35) | Germany  (refugees) | 100 (UPID) | 46.15 | UPID | 6 | 7–16  (11.5) | ITT | 5 |
| Santiago et al. (2018)  MDT (10 group +2 individual sessions, 50-60 min + 30-50min.)  WL | 25  27 | Bounce Back (group and individual, parent involvement) | Multiple  Types | Multiple*  (6.9) | USA | n.r. | 36.54 | UCLA PTSD-RI | 3 | 1st-4th grade (7.76) | ITT | 5 |
| Schauer (2008)  TF-CBT (6 sessions, 60-90 min.)  Meditation (6 sessions, 60-90 min.) | 25  22 | KIDNET  Meditation  (individual) | War | Multiple* (n.a.) | Sri Lanka | 100 (CAPS-CA) | 61.70 | CAPS-CA | 5 | 11-15  (13.1) | ITT | 7 |
| Scheeringa et al. (2011)  TF-CBT (12 sessions, 45 min.)  WL | 20  11 | CBT (individual, parent involvement) | Multiple types | n.r.  (n.r.) | USA | 24 (PAPA) | 33.80 | PAPA | r.b.i. | 3–6  (5.3) | Compl. | 3 |
| Schottelkorb et al. (2012)  TF-CBT (12-20 sessions, 90 min.)  CCPT (24 sessions, 30 min.) | 12  14 | TF-CBT  CCPT  (individual,  parent involvement in both conditions) | War | Multiple (n.a.) | USA  (refugees) | 58 (UPID) | 45.20 | UPID | n.a. | 6-13  (9.1) | Compl. | 4 |
| Shechtman & Mor (2010)  Other (10 sessions, n.r.)  WL | 84  52 | ESGI  (group) | War and parental  loss | n.r.  (n.r.) | Israel | n.r. (CPTS-RI) | 68.00 | CPTS-RI | n.a. | 9-14 | Compl. | 5 |
| Shein-Szydlo et al. (2016)  TF-CBT (12 sessions, 60 min.)  WL | 50  49 | CBT-TSC  (individual) | Multiple  types | Multiple (n.a.) | Mexico | 100 (DISC) | 63.64 | CPSS | r.b.i. | 12-19  (14.9) | ITT | 7 |
| Sloan et al. (2011)  TF-CBT (3 sessions, 20 min.)  Neutral Writing (3 sessions, 20 min.) | 21^b^  21^b^ | Written Exposure Therapy (individual) | Multiple  Types | n.r. (n.r.) | USA | 100 (PSS-I) | n.r. | PSS-I | 1 | 18.90 (1.10) | Compl. | 5 |
| Smith et al. (2007)  TF-CBT (10 sessions, n.r.)  WL | 12  12 | TF-CBT  (individual, parent involvement) | Multiple types | Single (1) | UK | 100 (CAPS-CA) | 39.47 | CAPS-CA | n.a. | 8–18  (13.8) | ITT | 7 |
| Stein et al. (2003)  TF-CBT (10 sessions, 60 min.)  WL | 54  63 | TF-CBT  (group) | Multiple types | n.r. (8.75) | USA | n.r. | 56.35 | CPSS | n.a. | 6th grade (11.0) | Compl. | 6 |
| Trowell et al. (2002)  Other (30 sessions, 50 min.)  Psychoeducation (18 sessions, n.r.) | 28  28 | PDP (individual)  Psychoeducation (group) | Sexual assault | Multiple (n.a.) | UK | n.r. | 100 | K-SADS | 24 | 6–14  (10.0) | Compl. | 4 |

*Note*: ACC = Active Control Condition; AD = Aerobic Dance control group; ADIS = Anxiety Disorders Interview Schedule; CAPS-CA = Clinician Administered PTSD Scale for Children and Adolescents; CBT = Cognitive Behavior Therapy; CBT-TSC = CBT for trauma in street children; CBT-3M = Cognitive Behavior Therapy – 3 M; CCPT = Child Centered Play Therapy; CCT = Child Centered Therapy; CITES-R = Children's Impact of Traumatic Events Scales-Revised; Compl.= completer analysis; CPSS = Child PTSD Symptom Scale; CPSS-I = CPSS Interview version; CPT = Cognitive Processing Therapy; CPTS-RI = Child Post-Traumatic Stress - Reaction Index; CPTSDI = Child PTSD Inventory; CRI = Child Reaction Index; CRIES = Children's Revised Impact of Event Scale; CRTI = Children’s Responses to Trauma Inventory; CT = Cognitive Therapy; D-CPT = Developmentally adapted Cognitive Processing Therapy; DICA = Diagnostic Interview for Children and Adolescents; DISC = Diagnostic Interview Schedule for Children; DR Congo = Democratic Republic of Congo; EFT = Emotional Freedom Techniques; EMDR = Eye Movement Desensitization and Reprocessing; EMDR-IGTP-OTS = EMDR-Integrative Group Treatment Protocol for Ongoing Traumatic Stress; ESGI = Expressive Supportive Group Intervention; freq = frequency; FU = Follow-Up; HTQ = Harvard Trauma Questionnaire; ITT = intent-to-treat analysis; K-SADS = Schedule for Affective Disorders and Schizophrenia for School-Age Children; KIDNET = Narrative Exposure Therapy for Children; MDT = Multidisciplinary Treatment; MINI-KID = Mini International Neuropsychiatric Interview for Children and Adolescents; n.a. = not applicable/no follow-up assessment reported; NET = Narrative Exposure Therapy; NL = the Netherlands; n.r. = not reported (i.e., either not reported at all or not reported in sufficient detail); NST = Non-directive Supportive Therapy; Other = Other psychological intervention (i.e., non-TF-CBT, non-EMDR, & non-MDT); PAPA = The Preschool Age Psychiatric Assessment; PE = Prolonged Exposure; PDP = Psychodynamic Psychotherapy; PSS-SR = PTSD Symptom Scale, PST = Problem Solving Therapy; Self-Report; PTSD = Post-Traumatic Stress Disorder; PTSS-C = Posttraumatic Stress Symptoms Scale for Children; r.b.i. = reported but irrelevant (i.e., irrelevant as no meaningful comparison possible at follow-up due to crossover-design/delayed treatment control, due to subgroup n < 10 or due to incomplete data reporting); RRFT = Risk Reduction through Family Therapy; SC = Supportive Counselling; SF-AT = Solution-Focused Art Therapy; SPTSS = Scale of Posttraumatic Stress Symptoms; TARGET = Trauma Affect Regulation: Guide for Education and Therapy; TAU = Treatment as Usual; TF-CBT = Trauma-focused Cognitive Behavior Therapy; TF-CBT-c = TF-CBT for child only; TF-CBT-c&p = TF-CBT for child and parent; TF-CBT-p = TF-CBT for parent only; TRT = Teaching Recovery Techniques; TRT+ = Teaching Recovery Techniques including TF-CBT and EMDR techniques; TSCC = Trauma Symptom Checklist for Children; UCLA PTSD-RI = University of California–Los Angeles PTSD Reaction Index; UK = United Kingdom; UPID = The University of California at Los Angeles PTSD Index; USA = United States of America; WL = Waitlist; YRI = Youth Readiness Intervention. References of included trials can be found in the Appendix.
^a^Diagnostic interviews took place three weeks after a tsunami. Hence, a subgroup of participants did not meet the 4-weeks PTSD time criterion at baseline.
^b^Since only FU was assessed, FU one groups sizes are reported.
*Indicates that all included participants had experienced multiple trauma exposures. That is, the given trial was included in the sensitivity analysis comparing trials with ≥ 90% multiple trauma to 100% single trauma trials.

**Appendix D. References of included trials**

Ahmad, A., Larsson, B., & Sundelin Wahlsten, V. (2007). EMDR treatment for children with PTSD: Results of a randomized controlled trial. *Nordic Journal of Psychiatry*, *61*(5), 349–354.

Ahrens, J., & Rexford, L. (2002). Cognitive processing therapy for incarcerated adolescents with PTSD. *Journal of Aggression, Maltreatment & Trauma*, *6*(1), 201–216.

Al-Hadethe, A., Hunt, N., Ghaffar, A.-Q., & Thomas, S. A. (2015). Randomised controlled study comparing two psychological therapies for posttraumatic stress disorder (PTSD): Emotional Freedom Techniques (EFT) vs. Narrative Exposure Therapy (NET). *Journal of Traumatic Stress Disorders & Treatment*, *4*(4).

Auslander, W., Edmond, T., Foster, A., Smith, P., McGinnis, H., Gerke, D., . . . Dunn, J. (2020). Cognitive behavioral intervention for trauma in adolescent girls in child welfare: A randomized controlled trial. *Children and Youth Services Review*, *119*.

Auslander, W., McGinnis, H., Tlapek, S., Smith, P., Foster, A., Edmond, T., & Dunn, J. (2017). Adaptation and implementation of a trauma-focused cognitive behavioral intervention for girls in child welfare. *American Journal of Orthopsychiatry*, *87*(3), 206–215.

Barron, I., Abdallah, G., & Heltne, U. (2016). Randomized control trial of teaching recovery techniques in rural occupied Palestine: Effect on adolescent dissociation. *Journal of Aggression, Maltreatment & Trauma*, *25*(9), 955–973.

Barron, I., Freitas, F., & Bosch, C. A. (2021). Pilot randomized control trial: Efficacy of a group-based psychosocial program for youth with PTSD in the Brazilian favelas. *Journal of Child & Adolescent Trauma*, *14*(3), 335–345.

Carrion, V. G., Kletter, H., Weems, C. F., Berry, R. R., & Rettger, J. P. (2013). Cue‐centered treatment for youth exposed to interpersonal violence: A randomized controlled trial. *Journal of Traumatic Stress*, *26*(6), 654–662.

Catani, C., Kohiladevy, M., Ruf, M., Schauer, E., Elbert, T., & Neuner, F. (2009). Treating children traumatized by war and tsunami: A comparison between exposure therapy and meditation-relaxation in North-East Sri Lanka. *BMC Psychiatry*, *9*, 1–11.

Celano, M., Hazzard, A., Webb, C., & McCall, C. (1996). Treatment of traumagenic beliefs among sexually abused girls and their mothers: An evaluation study. *Journal of Abnormal Child Psychology*, *24*(1), 1–17.

Chemtob, C. M., Nakashima, J., & Carlson, J. G. (2002). Brief treatment for elementary school children with disaster-related posttraumatic stress disorder: A field study. *Journal of Clinical Psychology*, *58*(1), 99–112.

Chen, Y., Shen, W. W., Gao, K., Lam, C. S., Chang, W. C., & Deng, H. (2014). Effectiveness RCT of a CBT intervention for Youths who lost parents in the Sichuan, China, earthquake. *Psychiatric Services*, *65*(2), 259–262.

Cohen, J. A., Mannarino, A. P., & Iyengar, S. (2011). Community treatment of posttraumatic stress disorder for children exposed to intimate partner violence: A randomized controlled trial. *Archives of Pediatrics & Adolescent Medicine*, *165*(1), 16–21.

Cohen, J. A., Mannarino, A. P., & Knudsen, K. (2005). Treating sexually abused children: 1 year follow-up of a randomized controlled trial. *Child Abuse & Neglect*, *29*(2), 135–145.

Cohen, J. A., Deblinger, E., Mannarino, A. P., & Steer, R. A. (2004). A multisite, randomized controlled trial for children with sexual abuse–related PTSD symptoms. *Journal of the American Academy of Child & Adolescent Psychiatry*, *43*(4), 393–402.

Danielson, C. K., McCart, M. R., Walsh, K., Arellano, M. A. de, White, D., & Resnick, H. S. (2012). Reducing substance use risk and mental health problems among sexually assaulted adolescents: A pilot randomized controlled trial. *Journal of Family Psychology*, *26*(4), 628–635.

Dawson, K., Joscelyne, A., Meijer, C., Steel, Z., Silove, D., & Bryant, R. A. (2018). A controlled trial of trauma-focused therapy versus problem-solving in Islamic children affected by civil conflict and disaster in Aceh, Indonesia. *Australian & New Zealand Journal of Psychiatry*, *52*(3), 253–261.

Deblinger, E., Lippmann, J., & Steer, R. (1996). Sexually abused children suffering posttraumatic stress symptoms: Initial treatment outcome findings. *Child Maltreatment*, *1*(4), 310–321.

Diehle, J., Opmeer, B. C., Boer, F., Mannarino, A. P., & Lindauer, R. J. L. (2015). Trauma-focused cognitive behavioral therapy or eye movement desensitization and reprocessing: What works in children with posttraumatic stress symptoms? A randomized controlled trial. *European Child & Adolescent Psychiatry*, *2*(24), 227–236.

Dorsey, S., Lucid, L., Martin, P., King, K. M., O’Donnell, K., Murray, L. K., . . . Manongi, R. (2020). Effectiveness of task-shifted trauma-focused cognitive behavioral therapy for children who experienced parental death and posttraumatic stress in Kenya and Tanzania: A randomized clinical trial. *JAMA Psychiatry*, *77*(5), 464–473.

Ertl, V., Pfeiffer, A., Schauer, E., Elbert, T., & Neuner, F. (2011). Community-implemented trauma therapy for former child soldiers in Northern Uganda: A randomized controlled trial. *JAMA*, *306*(5), 503–512.

Foa, E. B., McLean, C. P., Capaldi, S., & Rosenfield, D. (2013). Prolonged exposure vs supportive counseling for sexual abuse-related PTSD in adolescent girls: A randomized clinical trial. *JAMA*, *310*(24), 2650–2657.

Ford, J. D., Steinberg, K. L., Hawke, J., Levine, J., & Zhang, W. (2012). Randomized trial comparison of emotion regulation and relational psychotherapies for PTSD with girls involved in delinquency. *Journal of Clinical Child & Adolescent Psychology*, *41*(1), 27–37.

Gilboa-Schechtman, E., Foa, E. B., Shafran, N., Aderka, I. M., Powers, M. B., Rachamim, L., . . . Apter, A. (2010). Prolonged exposure versus dynamic therapy for adolescent PTSD: A pilot randomized controlled trial. *Journal of the American Academy of Child & Adolescent Psychiatry*, *49*(10), 1034–1042.

Goldbeck, L., Muche, R., Sachser, C., Tutus, D., & Rosner, R. (2016). Effectiveness of trauma-focused cognitive behavioral therapy for children and adolescents: A randomized controlled trial in eight German mental health clinics. *Psychotherapy and Psychosomatics*, *85*(3), 159–170.

Gordon, J. S., Staples, J. K., Blyta, A., Bytyqi, M., & Wilson, A. T. (2008). Treatment of posttraumatic stress disorder in postwar Kosovar adolescents using mind-body skills groups: A randomized controlled trial. *Journal of Clinical Psychiatry*, *69*(9), 1469–1476.

Hitchcock, C., Goodall, B., Wright, I. M., Boyle, A., Johnston, D., Dunning, D., . . . McKinnon, A. (2022). The early course and treatment of posttraumatic stress disorder in very young children: Diagnostic prevalence and predictors in hospital‐attending children and a randomized controlled proof‐of‐concept trial of trauma‐focused cognitive therapy, for 3‐to 8‐year‐olds. *Journal of Child Psychology and Psychiatry*, *63*(1), 58–67.

Jensen, T. K., Holt, T., Ormhaug, S. M., Egeland, K., Granly, L., Hoaas, L. C., . . . Wentzel-Larsen, T. (2014). A randomized effectiveness study comparing trauma-focused cognitive behavioral therapy with therapy as usual for youth. *Journal of Clinical Child & Adolescent Psychology*, *43*(3), 356–369.

Kameoka, S., Tanaka, E., Yamamoto, S., Saito, A., Narisawa, T., Arai, Y., . . . Asukai, N. (2020). Effectiveness of trauma-focused cognitive behavioral therapy for Japanese children and adolescents in community settings: A multisite randomized controlled trial. *European Journal of Psychotraumatology*, *11*(1), 1767987.

Kemp, M., Drummond, P., & McDermott, B. (2010). A wait-list controlled pilot study of eye movement desensitization and reprocessing (EMDR) for children with post-traumatic stress disorder (PTSD) symptoms from motor vehicle accidents. *Clinical Child Psychology and Psychiatry*, *15*(1), 5–25.

King, N. J., Tonge, B. J., Mullen, P., Myerson, N., Heyne, D., Rollings, S., . . . Ollendick, T. H. (2000). Treating sexually abused children with posttraumatic stress symptoms: A randomized clinical trial. *Journal of the American Academy of Child & Adolescent Psychiatry*, *39*(11), 1347–1355.

Langley, A. K., Gonzalez, A., Sugar, C. A., Solis, D., & Jaycox, L. (2015). Bounce back: Effectiveness of an elementary school-based intervention for multicultural children exposed to traumatic events. *Journal of Consulting and Clinical Psychology*, *83*(5), 853–865.

Lesmana, C. B.J., Suryani, L. K., Jensen, G. D., & Tiliopoulos, N. (2009). A spiritual-hypnosis assisted treatment of children with PTSD after the 2002 Bali terrorist attack. *American Journal of Clinical Hypnosis*, *52*(1), 23–34.

McMullen, J., O'callaghan, P., Shannon, C., Black, A., & Eakin, J. (2013). Group trauma‐focused cognitive‐behavioural therapy with former child soldiers and other war‐affected boys in the DR Congo: A randomised controlled trial. *Journal of Child Psychology and Psychiatry*, *54*(11), 1231–1241.

Meentken, M. G., van der Mheen, M., van Beynum, I. M., Aendekerk, E. W. C., Legerstee, J. S., van der Ende, J., . . . Moll, H. A. (2020). EMDR for children with medically related subthreshold PTSD: Short-term effects on PTSD, blood-injection-injury phobia, depression and sleep. *European Journal of Psychotraumatology*, *11*(1), 1705598.

Meiser‐Stedman, R., Smith, P., McKinnon, A., Dixon, C., Trickey, D., Ehlers, A., . . . Goodyer, I. (2017). Cognitive therapy as an early treatment for post‐traumatic stress disorder in children and adolescents: A randomized controlled trial addressing preliminary efficacy and mechanisms of action. *Journal of Child Psychology and Psychiatry*, *58*(5), 623–633.

Molero, R. J., Jarero, I., & Givaudan, M. (2019). Longitudinal multisite randomized controlled trial on the provision of the EMDR-IGTP-OTS to refugee minors in Valencia, Spain. *American Journal of Applied Psychology*, *8*(4), 77–88.

Murray, L. K., Skavenski, S., Kane, J. C., Mayeya, J., Dorsey, S., Cohen, J. A., . . . Bolton, P. A. (2015). Effectiveness of trauma-focused cognitive behavioral therapy among trauma-affected children in Lusaka, Zambia: A randomized clinical trial. *JAMA Pediatrics*, *169*(8), 761–769.

O’Callaghan, P., McMullen, J., Shannon, C., Rafferty, H., & Black, A. (2013). A randomized controlled trial of trauma-focused cognitive behavioral therapy for sexually exploited, war-affected Congolese girls. *Journal of the American Academy of Child & Adolescent Psychiatry*, *52*(4), 359–369.

O'callaghan, P., McMullen, J., Shannon, C., & Rafferty, H. (2015). Comparing a trauma focused and non trauma focused intervention with war affected Congolese youth: A preliminary randomised trial. *Intervention*, *13*(1), 28–44.

Osorio, A., Pérez, M. C., Tirado, S. G., Jarero, I., & Givaudan, M. (2018). Randomized controlled trial on the EMDR integrative group treatment protocol for ongoing traumatic stress with adolescents and young adults patients with cancer. *American Journal of Applied Psychology*, *7*(4), 50–56.

Peltonen, K., & Kangaslampi, S. (2019). Treating children and adolescents with multiple traumas: A randomized clinical trial of narrative exposure therapy. *European Journal of Psychotraumatology*, *10*(1), 1558708.

Pityaratstian, N., Piyasil, V., Ketumarn, P., Sitdhiraksa, N., Ularntinon, S., & Pariwatcharakul, P. (2014). Randomized controlled trial of group cognitive behavioural therapy for post-traumatic stress disorder in children and adolescents exposed to tsunami in Thailand. *Behavioural and Cognitive Psychotherapy*, *43*(5), 549–561.

Roos, C. de, Greenwald, R., den Hollander-Gijsman, M., Noorthoorn, E., van Buuren, S., & Jongh, A. de (2011). A randomised comparison of cognitive behavioural therapy (CBT) and eye movement desensitisation and reprocessing (EMDR) in disaster-exposed children. *European Journal of Psychotraumatology*, *2*(1), 5694.

Roos, C. de, van der Oord, S., Zijlstra, B., Lucassen, S., Perrin, S., Emmelkamp, P., & Jongh, A. D. de (2017). Comparison of eye movement desensitization and reprocessing therapy, cognitive behavioral writing therapy, and wait‐list in pediatric posttraumatic stress disorder following single‐incident trauma: A multicenter randomized clinical trial. *Journal of Child Psychology and Psychiatry*, *58*(11), 1219–1228.

Roque-Lopez, S., Llanez-Anaya, E., Álvarez-López, M. J., Everts, M., Fernández, D., Davidson, R. J., & Kaliman, P. (2021). Mental health benefits of a 1-week intensive multimodal group program for adolescents with multiple adverse childhood experiences. *Child Abuse & Neglect*, *122*, 105349.

Rosner, R., Rimane, E., Frick, U., Gutermann, J., Hagl, M., Renneberg, B., . . . Steil, R. (2019). Effect of developmentally adapted cognitive processing therapy for youth with symptoms of posttraumatic stress disorder after childhood sexual and physical abuse: A randomized clinical trial. *JAMA Psychiatry*, *76*(5), 484–491.

Rossouw, J., Yadin, E., Alexander, D., & Seedat, S. (2022). Long-term follow-up of a randomised controlled trial of prolonged exposure therapy and supportive counselling for post-traumatic stress disorder in adolescents: A task-shifted intervention. *Psychological Medicine*, *52*(6), 1022–1030.

Ruf, M., Schauer, M., Neuner, F., Catani, C., Schauer, E., & Elbert, T. (2010). Narrative exposure therapy for 7‐to 16‐year‐olds: A randomized controlled trial with traumatized refugee children. *Journal of Traumatic Stress*, *23*(4), 437–445.

Santiago, C. D., Raviv, T., Ros, A. M., Brewer, S. K., Distel, L. M. L., Torres, S. A., . . . Cicchetti, C. (2018). Implementing the Bounce Back trauma intervention in urban elementary schools: A real-world replication trial. *School Psychology Quarterly*, *33*(1), 1–9.

Schauer, E. (2008). *Trauma treatment for children in war: Build-up of an evidence-based large-scale mental health intervention in north-eastern Sri Lanka* (Doctoral dissertation).

Scheeringa, M. S., Weems, C. F., Cohen, J. A., Amaya‐Jackson, L., & Guthrie, D. (2011). Trauma‐focused cognitive‐behavioral therapy for posttraumatic stress disorder in three‐through six year‐old children: A randomized clinical trial. *Journal of Child Psychology and Psychiatry*, *52*(8), 853–860.

Schottelkorb, A. A., Doumas, D. M., & Garcia, R. (2012). Treatment for childhood refugee trauma: A randomized, controlled trial. *International Journal of Play Therapy*, *21*(2), 57–73.

Shechtman, Z., & Mor, M. (2010). Groups for children and adolescents with trauma-related symptoms: Outcomes and processes. *International Journal of Group Psychotherapy*, *60*(2), 221–244.

Shein‐Szydlo, J., Sukhodolsky, D. G., Kon, D. S., Tejeda, M. M., Ramirez, E., & Ruchkin, V. (2016). A randomized controlled study of cognitive–behavioral therapy for posttraumatic stress in street children in Mexico City. *Journal of Traumatic Stress*, *29*(5), 406–414.

Sloan, D. M., Marx, B. P., & Greenberg, E. M. (2011). A test of written emotional disclosure as an intervention for posttraumatic stress disorder. *Behaviour Research and Therapy*, *49*(4), 299–304.

Smith, P., Yule, W., Perrin, S., Tranah, T., Dalgleish, T. I.M., & Clark, D. M. (2007). Cognitive-behavioral therapy for PTSD in children and adolescents: A preliminary randomized controlled trial. *Journal of the American Academy of Child & Adolescent Psychiatry*, *46*(8), 1051–1061.

Stein, B. D., Jaycox, L. H., Kataoka, S. H., Wong, M., Tu, W., Elliott, M. N., & Fink, A. (2003). A mental health intervention for schoolchildren exposed to violence: A randomized controlled trial. *JAMA*, *290*(5), 603–611.

Trowell, J., Kolvin, I., Weeramanthri, T., Sadowski, H., Berelowitz, M., Glasser, D., & Leitch, I. (2002). Psychotherapy for sexually abused girls: Psychopathological outcome findings and patterns of change. *The British Journal of Psychiatry*, *180*(3), 234–247.

**Appendix E. Trial quality of included trials**

| Trial / reference | Q1 – 100% PTSD rate at baseline | Q2 -manual-based treatment | Q3 – therapists trained in manual | Q4 -integrity/ manual adherence checked | Q5 – intent-to-treat (ITT) results reported | Q6 –  N > 50 | Q7 -independent randomization | Q8 - blinded outcome assessment | Q sum score  (out of 8) |
| --- | --- | --- | --- | --- | --- | --- | --- | --- | --- |
| Ahmad et al. (2007) | 1 | 1 | 0 | 0 | 1 | 0 | 0 | 1 | 4 |
| Ahrens & Rexford (2002) | 0 | 1 | 1 | 0 | 0 | 0 | 0 | 1 | 3 |
| Al-Hadethe et al. (2015) | 0 | 0 | 0 | 0 | 0 | 0 | 1 | 1 | 2 |
| Auslander et al. (2017) | 0 | 1 | 1 | 1 | 0 | 0 | 1 | 1 | 5 |
| Auslander et al. (2020) | 0 | 1 | 1 | 1 | 1 | 1 | 1 | 0 | 6 |
| Barron et al. (2016) | 0 | 1 | 1 | 1 | 1 | 1 | 1 | 1 | 7 |
| Barron et al. (2020) | 0 | 1 | 1 | 1 | 1 | 0 | 0 | 1 | 5 |
| Carrion et al. (2013) | 0 | 1 | 1 | 1 | 1 | 1 | 1 | 1 | 7 |
| Catani et al. (2009) | 0 | 1 | 1 | 1 | 1 | 0 | 1 | 1 | 6 |
| Catani et al. (2009) | 0 | 1 | 1 | 1 | 1 | 0 | 1 | 1 | 6 |
| Celano et al. (1996) | 0 | 1 | 1 | 1 | 0 | 0 | 0 | 1 | 4 |
| Chemtob et al. (2002) | 1 | 1 | 1 | 1 | 0 | 0 | 0 | 1 | 5 |
| Chen et al. (2014) | 0 | 1 | 0 | 0 | 0 | 0 | 0 | 1 | 2 |
| Cohen et al. (2004) | 0 | 1 | 1 | 1 | 0 | 1 | 0 | 1 | 5 |
| Cohen et al. (2005) | 0 | 1 | 0 | 1 | 1 | 1 | 1 | 1 | 6 |
| Cohen et al. (2011) | 0 | 1 | 1 | 1 | 1 | 1 | 1 | 1 | 7 |
| Danielson et al. (2012) | 0 | 0 | 1 | 1 | 1 | 0 | 1 | 1 | 5 |
| Dawson et al. (2018) | 0 | 0 | 1 | 0 | 1 | 1 | 1 | 1 | 5 |
| de Roos et al. (2011) | 0 | 1 | 1 | 1 | 1 | 0 | 1 | 1 | 6 |
| de Roos et al. (2017) | 0 | 1 | 1 | 1 | 1 | 1 | 1 | 1 | 7 |
| Deblinger et al. (1996) | 0 | 1 | 1 | 1 | 0 | 0 | 0 | 0 | 3 |
| Diehle et al. (2015) | 0 | 1 | 1 | 1 | 1 | 0 | 1 | 1 | 6 |
| Dorsey et al. (2020) | 0 | 1 | 1 | 1 | 1 | 1 | 1 | 1 | 7 |
| Ertl et al. (2011) | 1 | 1 | 1 | 1 | 0 | 1 | 0 | 1 | 6 |
| Foa et al. (2013) | 0 | 1 | 1 | 1 | 1 | 1 | 1 | 1 | 7 |
| Ford et al. (2012) | 0 | 1 | 1 | 1 | 1 | 1 | 1 | 0 | 6 |
| Gilboa-Schechtman et al. (2010) | 1 | 1 | 1 | 1 | 1 | 0 | 1 | 1 | 7 |
| Goldbeck et al. (2016) | 0 | 1 | 1 | 1 | 1 | 1 | 1 | 1 | 7 |
| Gordon et al. (2008) | 0 | 1 | 1 | 0 | 1 | 1 | 1 | 1 | 6 |
| Hitchcock et al. (2021) study 2 | 1 | 1 | 1 | 1 | 1 | 0 | 1 | 1 | 7 |
| Jensen et al. (2014) | 0 | 1 | 1 | 1 | 1 | 1 | 1 | 1 | 7 |
| Kameoka et al. (2020) | 0 | 1 | 1 | 1 | 1 | 0 | 1 | 1 | 6 |
| Kemp et al. (2010) | 0 | 1 | 1 | 1 | 0 | 0 | 0 | 0 | 3 |
| King et al. (2000) | 0 | 1 | 1 | 1 | 1 | 0 | 0 | 0 | 4 |
| Langley et al. (2015) | 0 | 1 | 1 | 1 | 0 | 1 | 1 | 1 | 6 |
| Lesmana et al. (2009) | 0 | 0 | 0 | 0 | 1 | 1 | 0 | 0 | 2 |
| McMullen et al. (2013) | 0 | 1 | 1 | 1 | 0 | 0 | 1 | 1 | 5 |
| Meentken et al. (2020) | 0 | 1 | 0 | 1 | 1 | 1 | 1 | 1 | 6 |
| Meiser-Stedman et al. (2017) | 1 | 1 | 1 | 1 | 0 | 0 | 0 | 1 | 5 |
| Molero et al. (2019) | 0 | 1 | 1 | 0 | 0 | 1 | 1 | 1 | 5 |
| Murray et al. (2015) | 0 | 1 | 1 | 1 | 1 | 1 | 1 | 1 | 7 |
| O'Callaghan et al. (2013) | 0 | 1 | 1 | 1 | 1 | 1 | 1 | 1 | 7 |
| O'Callaghan et al. (2015) | 0 | 1 | 1 | 1 | 1 | 1 | 1 | 1 | 7 |
| Osorio et al. (2018) | 0 | 1 | 1 | 1 | 1 | 0 | 1 | 1 | 6 |
| Peltonen et al. (2019) | 0 | 1 | 1 | 1 | 0 | 0 | 1 | 0 | 4 |
| Pityaratstian et al. (2015) | 1 | 1 | 1 | 0 | 0 | 0 | 0 | 1 | 4 |
| Roque-Lopez et al. (2021) | 0 | 1 | 1 | 0 | 0 | 0 | 1 | 0 | 3 |
| Rosner et al. (2019) | 1 | 1 | 1 | 1 | 1 | 1 | 1 | 1 | 8 |
| Rossouw et al. (2020) | 1 | 1 | 1 | 1 | 1 | 1 | 1 | 1 | 8 |
| Ruf et al. (2010) | 1 | 1 | 1 | 0 | 1 | 0 | 0 | 1 | 5 |
| Santiago et al. (2018) | 0 | 1 | 1 | 0 | 1 | 1 | 0 | 1 | 5 |
| Schauer (2008) | 1 | 1 | 1 | 1 | 1 | 0 | 1 | 1 | 7 |
| Scheeringa et al. (2011) | 0 | 1 | 0 | 1 | 0 | 0 | 1 | 0 | 3 |
| Schottelkorb et al. (2012) | 0 | 1 | 1 | 1 | 0 | 0 | 1 | 0 | 4 |
| Shechtman & Mor (2010) | 0 | 1 | 1 | 1 | 0 | 1 | 0 | 1 | 5 |
| Shein-Szydlo et al. (2016) | 1 | 1 | 1 | 1 | 0 | 1 | 1 | 1 | 7 |
| Sloan et al. (2011) | 1 | 1 | 1 | 1 | 0 | 0 | 0 | 1 | 5 |
| Smith et al. (2007) | 1 | 1 | 1 | 1 | 1 | 0 | 1 | 1 | 7 |
| Stein et al. (2003) | 0 | 1 | 1 | 1 | 0 | 1 | 1 | 1 | 6 |
| Trowell et al. (2002) | 0 | 1 | 1 | 1 | 0 | 1 | 0 | 0 | 4 |

**Appendix F. Forest plot depicting the efficacy of trauma-focused cognitive behavior therapy vs. passive control conditions at treatment endpoint in samples exposed to a single trauma (top) or multiple traumas (bottom)**


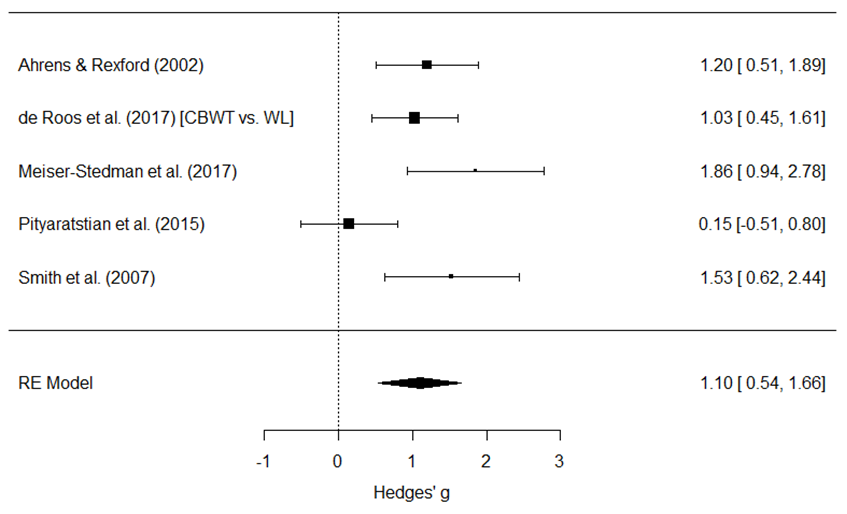


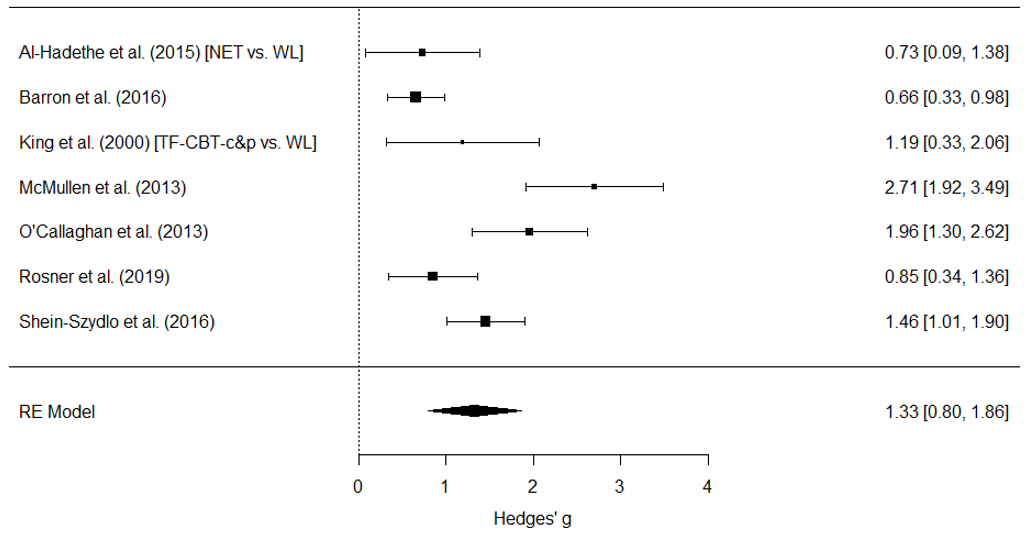


Abbreviations. NET = Narrative Exposure Therapy; TF-CBT-c&p = Trauma-focused cognitive behavior therapy treatment arm with parent involvement; WL = Waitlist control condition. Note that square brackets behind the reference indicate that the given trial had more than two relevant arms. Brackets contain the extracted (primary) comparison. Other comparisons were neglected to avoid data dependencies.

**Appendix G. Forest plots depicting the efficacy of trauma-focused cognitive behavior therapy vs. other psychological interventions at treatment endpoint in samples exposed to a single trauma (top) or multiple traumas (bottom)**


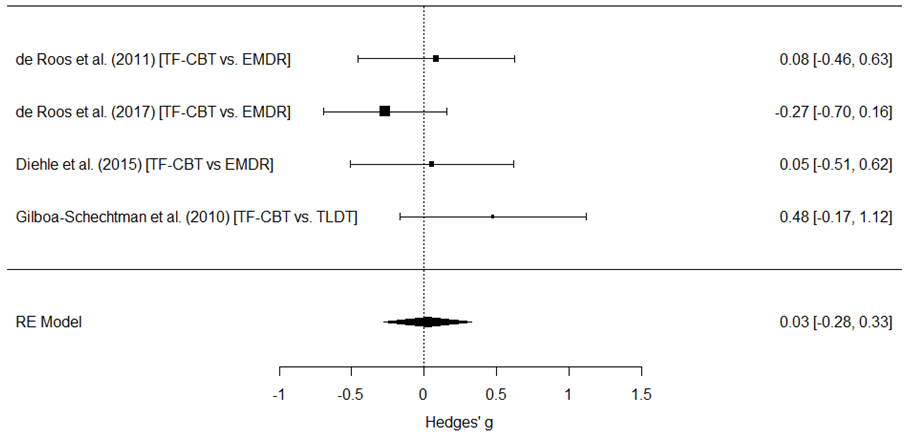


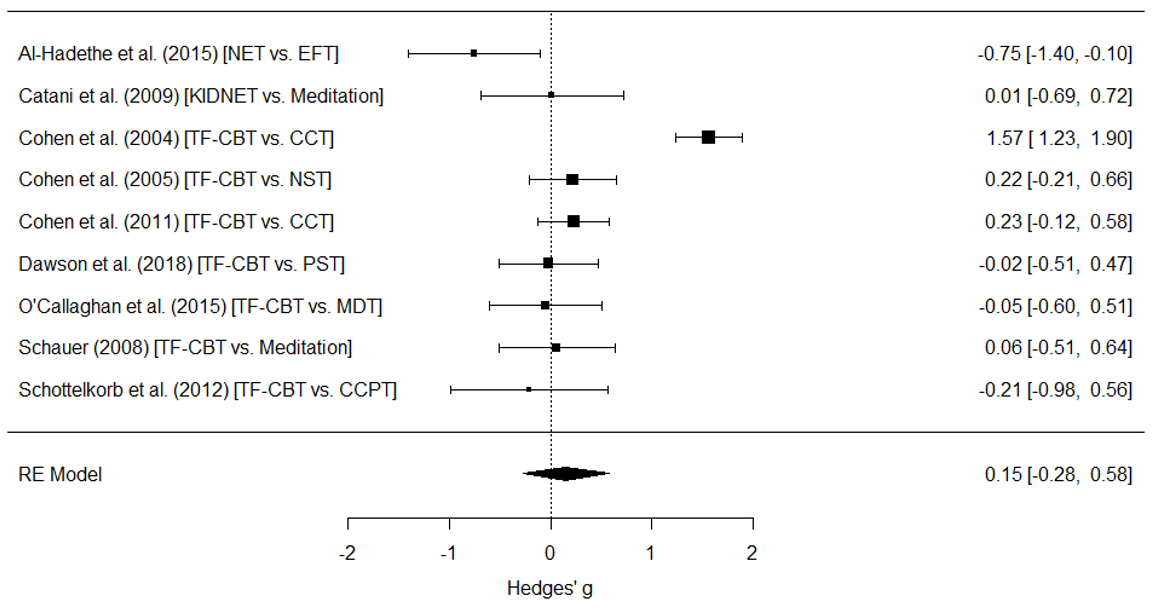


Abbreviations. CCPT = Child Centered Play Therapy; CCT = Child Centered Therapy; EFT = Emotional Freedom Techniques; KIDNET = Narrative Exposure Therapy for refugee children (specialized treatment protocol); MDT = Multi-Disciplinary Treatment; NET = Narrative Exposure Therapy; NST = Non-directive Supportive Therapy; PST = Problem Solving Therapy; TF-CBT = Trauma-focused cognitive behavior therapy treatment; TLDT = Time Limited Dynamic Therapy.

**Appendix H.** **Sensitivity analysis^1^: Efficacy of psychological interventions for pediatric PTSD for single vs. multiple trauma trials at posttreatment**

| **Comparison** | ***Single vs. multiple (trauma) exposures*** | ***k*** | ***g*** | **95% CI**  **(PI)** | ***I*²** | **NNT** | ***Moderation test, p*** |
| --- | --- | --- | --- | --- | --- | --- | --- |
| All interventions vs. passive control conditions | single | 8 | **1.09***** | **0.70 – 1.48 (0.20 – 1.98)** | 52.88* | 1.79 | .768 |
|  | multiple | 9 | 1.19*** | 0.75 – 1.64 (-0.05 – 2.44) | 80.70*** | 1.66 |  |
| All interventions vs. active control conditions | single | n.a. (*k* = 1) | | | | | n.a. |
|  | multiple | 8 | 0.49** | 0.12 – 0.85 (-0.43 – 1.41) | 75.72*** | 3.71 |  |
| TF-CBT vs. passive control conditions | single | 5 | 1.10*** | 0.54 – 1.66 (-0.06 – 2.26) | 65.99* | 1.78 | .667 |
|  | multiple | 6 | 1.32*** | 0.67 – 1.96 (-0.26 – 2.89) | 86.24*** | 1.54 |  |
| TF-CBT vs. active control conditions | single | n.a. (*k* = 1) | | | | | n.a. |
|  | multiple | 5 | 0.53** | 0.13 – 0.93 (-0.30 – 1.37) | 75.10*** | 3.41 |  |
| EMDR vs. passive control conditions | single | 4 | **1.11***** | **0.72 – 1.50 (0.65 – 1.58)** | 9.99 | 1.76 | n.a. |
|  | multiple | n.a. (*k* = 0) | | | | |  |
| TF-CBT vs. other psychological interventions (EMDR, MDTs) | single | 4 | 0.03 | -0.28 – 0.33 (-0.39 – 0.45) | 22.38 | 63.53 | .772 |
|  | multiple | 5 | 0.20 | -0.58 – 0.97 (-1.62 – 2.01) | 90.41*** | 9.04 |  |

Abbreviations. ACC = Active Control Conditions; EMDR = Eye Movement Desensitization and Reprocessing; *k* = number of trials included in the analysis for the given comparison; MDT = Multi-Disciplinary Treatments (i.e., involving a mixture of techniques from at least two families such as TF-CBT techniques + EMDR techniques); n.a. = not applicable (i.e., number of trials too small [k < 4] to conduct analysis); PCC = Passive Control Conditions; PI = Prediction Interval; TF-CBT = Trauma-focused Cognitive Behavioral Therapy;. *p*-values refer to the statistical significance level of the respective analyzed moderator. **Bold** font indicates that the CI as well as the PI exclude the null highlighting large certainty in the respective efficacy.
^1^More conservative definition applied for *multiple trauma trial* (i.e., ≥ 90% of sample reported exposure to ≥ 2 traumas rather than ≥ 50 % in the main analysis).
* *p* < 0.05; ** *p* < 0.01; *** *p* < 0.001

**Appendix I. Long-term efficacy of psychological interventions for pediatric PTSD in samples exposed to a single trauma vs. multiple traumas**

| **Comparison** | ***Follow up time*** | ***Single vs. multiple (trauma) exposure*** | ***k*** | ***g*** | **95% CI**  **(PI)** | ***I*²** | **NNT** | **Moderator test, p** |
| --- | --- | --- | --- | --- | --- | --- | --- | --- |
| All interventions vs. passive control conditions | FU1 | single | n.a. (*k* = 2) | | | | | n.a. |
|  |  | multiple | 6 | 0.67** | 0.22 – 1.11 (-0.30 – 1.64) | 65.15* | 2.76 |  |
|  | FU2 |  | n.a. (*k* = 0) | | | | | n.a. |
|  |  |  | n.a. (*k* = 2) | | | | |  |
| All interventions vs. active control conditions | FU1 | single | n.a. (*k* = 1) | | | | | n.a. |
|  |  | multiple | 6 | 0.28 | -0.11 – 0.66 (-0.55 – 1.10) | 66.36* | 6.48 |  |
|  | FU2 | single | n.a. (*k* = 0) | | | | | n.a. |
|  |  | multiple | 6 | 0.49*** | 0.20 – 0.78 (-0.04 – 1.03) | 41.80 | 3.67 |  |
| TF-CBT vs. passive control conditions | FU1 | single | n.a. (*k* = 1) | | | | | n.a. |
|  |  | multiple | 5 | 0.45* | 0.08 – 0.81  (-0.19 – 1.08) | 41.35 | 4.04 |  |
|  | FU2 |  | n.a. (*k* = 0) | | | | | n.a. |
|  |  |  | n.a. (*k* = 2) | | | | |  |
| TF-CBT vs. active control conditions | FU1 | single | n.a. (*ks* < 4) | | | | | n.a. |
|  |  | multiple |  |  |  |  |  |  |
|  | FU2 |  | n.a. (*k* = 0) | | | | | n.a. |
|  |  |  | 4 | 0.58** | 0.22 – 0.94 (-0.05 – 1.21) | 51.32 | 3.15 |  |
| EMDR vs. passive control conditions | FU1 & FU2 | single | n.a. (*ks* < 4) | | | | | n.a. |
|  |  | multiple |  |  |  |  |  |  |
| TF-CBT vs. other psychological interventions (EMDR, MDTs) | FU1 | single | n.a. (*k* = 2) | | | | | n.a. |
|  |  | multiple | n.a. (*k* = 2) | | | | |  |
|  | FU2 | single | n.a. (*k* = 2) | | | | | n.a. |
|  |  | multiple | 5 | -0.05 | -0.46 – 0.37 (-0.92 – 0.83) | 70.58* | -37.72 |  |

Abbreviations. ACC = Active Control Conditions; EMDR = Eye Movement Desensitization and Reprocessing; FU1 = Follow-Up 1 (i.e., follow-up assessments ≤ 5 months after treatment completion and in case of multiple assessments the closest one to 5 months); FU2 = Follow-Up 2 (i.e., follow-up assessments > 5 months after treatment completion and in case of multiple assessments the longest assessment); *k* = number of trials included in the analysis for the given comparison; MDT = Multi-Disciplinary Treatments (i.e., involving a mixture of techniques from at least two families such as TF-CBT techniques + EMDR techniques); n.a. = not applicable (i.e., number of trials too small [k < 4] to conduct analysis); PCC = Passive Control Conditions; PI = Prediction Interval; TF-CBT = Trauma-focused Cognitive Behavioral Therapy;. *p*-values refer to the statistical significance level of the respective analyzed moderator.
* *p* < 0.05; ** *p* < 0.01; *** *p* < 0.001

**Appendix J. Trial quality as a potential moderator of short-term efficacy of psychological interventions for pediatric PTSD in samples exposed to a single trauma vs. multiple traumas**

| **Single vs. multiple trauma -**  **comparison** | ***Analyzed potential moderator*** | ***k*** | ***b*** | ***I*²** | ***p*** |
| --- | --- | --- | --- | --- | --- |
| **EFFICACY AT POSTTREATMENT** | | | | | |
| Single trauma - psychological interventions vs. passive control conditions | Trial quality | n.a. (*k* = 8) | | | |
|  |  |  |  |  |  |
|  |  |  |  |  |  |
|  |  |  |  |  |  |
| **EFFICACY AT POSTTREATMENT** | | | | | |
| Multiple traumas - psychological interventions vs. passive control conditions | Trial quality | 12 | 0.05 | 81.60*** | .636 |
|  |  |  |  |  |  |
|  |  |  |  |  |  |
| **EFFICACY AT POSTTREATMENT** | | | | | |
| Single trauma - psychological interventions vs. active control conditions | Trial quality | n.a. (*k* = 1) | | | |
|  |  |  |  |  |  |
|  |  |  |  |  |  |
|  |  |  |  |  |  |
| **EFFICACY AT POSTTREATMENT** | | | | | |
| Multiple traumas - psychological interventions vs. active control conditions | Trial quality | 14 | 0.02 | 77.95*** | .818 |
|  |  |  |  |  |  |
|  |  |  |  |  |  |

Abbreviations. *k* = number of trials included in the analysis for the given comparison; n.a. = not applicable (i.e., number of trials too small (k < 10) to conduct analysis). *p*-values refer to the statistical significance level of the respective analyzed moderator. Note that FU results are not listed since k < 10 for all analyses. Note that meta-regressions for follow-up data was too scarce (k < 10) to warrant analyses.
* *p* < 0.05; ** *p* < 0.01; *** *p* < 0.001
